# Endosomal Wnt signaling proteins control microtubule nucleation in dendrites

**DOI:** 10.1371/journal.pbio.3000647

**Published:** 2020-03-12

**Authors:** Alexis T. Weiner, Dylan Y. Seebold, Pedro Torres-Gutierrez, Christin Folker, Rachel D. Swope, Gregory O. Kothe, Jessica G. Stoltz, Madeleine K. Zalenski, Christopher Kozlowski, Dylan J. Barbera, Mit A. Patel, Pankajam Thyagarajan, Matthew Shorey, Derek M. R. Nye, Matthew Keegan, Kana Behari, Song Song, Jeffrey D. Axelrod, Melissa M. Rolls

**Affiliations:** 1 Department of Biochemistry and Molecular Biology, The Pennsylvania State University, University Park, Pennsylvania, United States of America; 2 Department of Pathology, Stanford University School of Medicine, Stanford, California, United States of America; Utrecht University, NETHERLANDS

## Abstract

Dendrite microtubules are polarized with minus-end-out orientation in *Drosophila* neurons. Nucleation sites concentrate at dendrite branch points, but how they localize is not known. Using *Drosophila*, we found that canonical Wnt signaling proteins regulate localization of the core nucleation protein γTubulin (γTub). Reduction of frizzleds (fz), arrow (low-density lipoprotein receptor-related protein [LRP] 5/6), dishevelled (dsh), casein kinase Iγ, G proteins, and Axin reduced γTub-green fluorescent protein (GFP) at branch points, and two functional readouts of dendritic nucleation confirmed a role for Wnt signaling proteins. Both dsh and Axin localized to branch points, with dsh upstream of Axin. Moreover, tethering Axin to mitochondria was sufficient to recruit ectopic γTub-GFP and increase microtubule dynamics in dendrites. At dendrite branch points, Axin and dsh colocalized with early endosomal marker Rab5, and new microtubule growth initiated at puncta marked with fz, dsh, Axin, and Rab5. We propose that in dendrites, canonical Wnt signaling proteins are housed on early endosomes and recruit nucleation sites to branch points.

## Introduction

Neurons extend long branched processes from a central cell body. This shape is incompatible with a centrosomal microtubule organizing center (MTOC). Mature neurons are therefore among the ranks of differentiated cells that have noncentrosomal microtubule arrays [1–4]. It is particularly important to understand how neuronal microtubules are organized because the distance from the primary site of synthesis in the cell body to functional sites in axons and dendrites can be large and, therefore, place heavy demands on microtubule-based transport. In humans, slight disruptions in microtubule regulators or motors can manifest as neurodegenerative disease [5, 6], underscoring neuronal reliance on perfectly orchestrated microtubule-based transport.

If neuronal microtubules are not anchored to the centrosome, how are they organized? In all neurons so far examined, axonal microtubules have their dynamic plus ends oriented away from the cell body (plus-end-out) [7]. In dendrites of vertebrate neurons, microtubules are mixed polarity [8–10]. In invertebrate neurons (*Drosophila* and *Caenorhabditis elegans*), axons have the same plus-end-out microtubule organization as vertebrates [11–14], but mature dendrites have almost all minus-end-out microtubules [11, 12, 14]. In immature *Drosophila* dendrites, microtubules are mixed polarity and only gradually resolve to the minus-end-out mature arrangement [15]. Thus, although the final arrangement of microtubules in vertebrate and invertebrate dendrites is somewhat different, they are the same during dendrite development.

Although the arrangement of neuronal microtubules is clearly noncentrosomal, the source of axonal and dendritic microtubules has been controversial. Two major models for generating axonal and dendritic microtubules have been proposed. The first is that neuronal microtubules are nucleated at the centrosome, or perhaps elsewhere in the cell body, and then released for transport/sliding into axons and dendrites [16]. This model has substantial support, including recent analyses with newer techniques. For example, live imaging of microtubules with plus-tip (+TIP) tracking proteins and photoconvertible αTubulin has provided evidence for directional transport of microtubules into and out of developing axons in mammalian [17] and *Drosophila* [18] neurons.

The second model is that nucleation sites are found outside the cell body and that microtubules are generated locally in axons and dendrites. Evidence for this model came from the observation that centrosomal γTubulin (γTub), the core microtubule nucleation protein, decreases gradually over time and that centrosome ablation does not disrupt axon formation [19]. Similarly, the centriole is not surrounded by γTub in *Drosophila* neurons in vivo, and it is dispensable for neuronal microtubule organization [20]. One way to reconcile these two models is to assume that both are important and that, very early in neuronal development, microtubule sliding can dominate, whereas later in development and in mature neurons, microtubules are primarily locally nucleated.

In some cell types, the Golgi complex recruits nucleation sites [21, 22], and small Golgi outposts can be found in both mammalian and *Drosophila* dendrites [23, 24]. Thus, it was proposed that the Golgi might act as a noncentrosomal MTOC in dendrites [25]. However, subsequent analysis of γTub and Golgi outposts, including a strategy to deplete Golgi from dendrites, called this proposal into question [26].

Within *Drosophila* dendrites, γTub is concentrated at branch points [26]. In a previous study, we identified proteins that localize a different microtubule regulator, adenomatous polyposis coli (Apc) 2, to branch points [27]. We reasoned that some or all of this machinery might be used to position γTub to the same region. We therefore tested whether any of the Apc2 localization proteins act upstream of γTub-green fluorescent protein (GFP) in dendrites. Surprisingly, a subset of Wnt signaling proteins was required to localize γTub-GFP to dendrite branch points, regulate dendritic microtubule polarity, and nucleate microtubules in dendrites in response to axon injury. The required proteins include the seven transmembrane domain frizzled (fz) proteins (Wnt receptors), arrow (arr, a Wnt coreceptor), heterotrimeric G proteins, dishevelled (dsh), casein kinase I (CK1)γ, and Axin. Axin seems to be the key output protein of this pathway because it was sufficient to recruit γTub to ectopic sites in dendrites. Within branch points, fz, Axin, and dsh were found on puncta that colocalized with Rab5. In addition, new end-binding protein 1 (EB1) comets at polymerizing microtubule plus ends initiated from puncta marked with fz, arr, dsh, Axin, and Rab5. We propose that Wnt signaling proteins localize to early endosomes at dendrite branch points and function there to control local microtubule nucleation. Although it has previously been shown that Wnt signaling proteins can function from endosomes [28], identification of microtubule nucleation as an output of endosomal Wnt proteins is quite unexpected.

## Results

### A subset of canonical Wnt signaling proteins is required for γTub concentration at dendrite branch points

**Fig 1:**
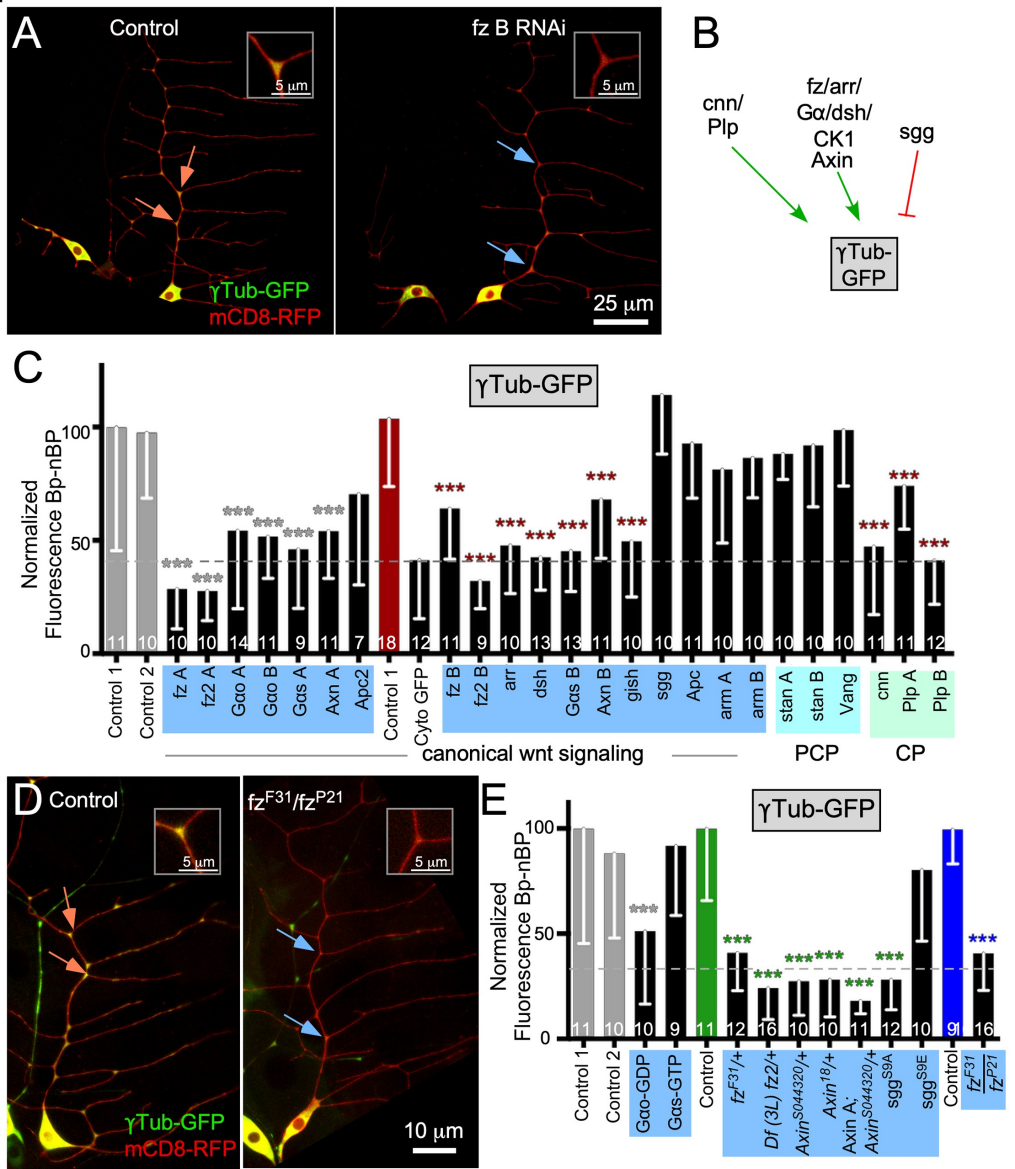
A candidate screen to identify proteins that target γTub-GFP to dendrite BPs. (A) Example images of UAS-γTub-GFP localization in ddaE neurons expressing UAS-Rtnl2 RNAi (control 1) (VDRC 33320) and UAS-fz B RNAi (VDRC 43075) hairpins. Membranes were marked with UAS-mCD8-RFP to see cell shape. Orange arrows indicate BPs with high γTub-GFP signal, and blue arrows indicate BPs with low γTub-GFP signal. Insets in the top corner of each image show the top BP highlighted with an arrow in each image (B) Diagram summarizing data in the figure is shown. See also S1 Fig for information about BP and nBP quantitation. (C) Quantification of γTub-GFP at BPs is shown in larvae expressing different RNAi hairpins. Control 1 is Rtnl2 RNAi because Rtnl2 is thought to be a pseudogene. Control 2 is γTub37C RNAi. This isoform of γTub is maternally deposited and not expressed in somatic cells like neurons. Values were generated by subtracting mean nBP fluorescence from BP fluorescence for each cell; normalized fluorescence values are shown. Normalization values were generated by making the control value equal to 100. The normalization constant was then used to normalize each sample from every other genotype. Additionally, each sample in the control is also normalized to this value. A dashed line indicates where a soluble GFP control is used to show baseline BP localization due to the difference in size and shape from nBB regions. This dashed line will continue throughout the rest of the figures that show γTub-GFP or Axin-GFP. Shaded colors over x-axis names indicate which functional groups the RNAi lines belong to and are noted as pink for mitochondria (S1 Fig), yellow for Ankyrin2 and Neuroglian (S1 Fig), purple for branched-actin regulators (S1 Fig), blue for wnt signaling, and green for centrosomal proteins. Color codes will follow throughout the rest of the figures. Gray bars are controls done on an Olympus FluoView1000; the same control data sets are shown in S1 Fig for reference. The red bars indicate a control performed on a Zeiss LSM800; the same control data set is shown in S1 Fig. (D) Example images of *yw* (control) and *fz*^*F31*^*/fz*^*P21*^ animals expressing UAS-γTub-GFP and UAS-mCD8-RFP with the promoter IGI-Gal4 to drive expression in class I da neurons. Orange arrows indicate BPs with high γTub-GFP signal, and blue arrows indicate BPs with low γTub-GFP signal. Insets in the top corner of each image indicate the top BP marked in each image. (E) Quantification of BP-nBP fluorescence as carried out before. Throughout the figures, data from the Olympus are on the left, and data from the Zeiss are on the right. Refer to S1 Table for all genotypes and S1 Data for data used to generate graphs in (C) and (E). Sample sizes are shown within the bars. Error bars indicate standard deviation. A linear regression was used to determine statistical significance. *p < 0.05, **p < 0.01, ***p < 0.001. γTub, γTubulin; Apc, adenomatous polyposis coli; arm, armadillo; arr, arrow; Axn, Axin; BP, branch point; CK1, casein kinase I; cnn, centrosomin; Cyto, cytoplasmic; da neuron; dendritic arborization neuron; dda, dorsal dendritic arborization; Df, deficiency; dsh, dishevelled; fz, frizzled; Gα, G protein alpha subunit; GFP, green fluorescent protein; gish, gilgamesh; nBP, non–branch point; Plp, Pericentrin-like protein; RFP, red fluorescent protein; RNAi, RNA interference; Rtnl2, reticulon 2; sgg, shaggy; stan, starry night; UAS, upstream activating sequence; Vang, Van Gogh; VDRC, Vienna Drosophila Resource Center; *yw*, *yellow*, *white*.

To understand how γTub is concentrated at dendrite branch points, we expressed γTub-GFP in a model *Drosophila* cell, the dorsal dendritic arborization (dda) E neuron. γTub-GFP localizes similarly to endogenous γTub [26] and can rescue phenotypes in mutant animals [29]. The ddaE cell is found in the larval body wall, where it helps sense body position to facilitate coordinated movement [30]. Microtubule organization in this cell type has been described in previous studies [12, 26, 31, 32], and the stereotyped shape of its large dorsal dendrite makes it easy to consistently assay protein localization. A soluble protein is about 1.2-fold brighter at branch points than at non–branch points, whereas γTub-GFP is over 2-fold brighter in this region [26], indicating that it is likely to be actively recruited. We therefore constructed a tester line, upstream activating sequence (UAS)-dicer2, UAS-mCD8-red fluorescent protein (RFP); 221-Gal4, UAS-γTub-GFP (Fig 1A), in which we could perform RNA interference (RNAi) and assay γTub-GFP in whole, living larvae. When this tester line was crossed to a control RNAi line, GFP fluorescence was higher within branch points than between them (Figs 1A and S1). To make sure that this represented active targeting rather than a larger volume of branch points, we compared γTub-GFP signal to cytoplasmic GFP signal (S1 Fig). Although cytoplasmic GFP signal is slightly higher at branch points than non–branch points, γTub-GFP is much higher (S1G Fig). The difference between cytoplasmic GFP at branch points versus non–branch points represents the lowest expected value for γTub-GFP when active targeting is removed and is indicated by a dotted line on graphs (Fig 1C and 1E). The γTub-GFP tester line was validated as a screening tool by crossing it to lines with RNAi transgenes targeting centrosomin (cnn) and Pericentrin-like protein (Plp), proteins previously implicated in dendritic microtubule nucleation [25, 32]. Compared with control, cnn and Plp RNAi resulted in less γTub-GFP at branch points (Fig 1C). Note that these effects are cell autonomous, as UAS-RNAi hairpins were expressed with 221-Gal4 in a small subset of neurons that includes ddaE.

The branch point localization of γTub-GFP is reminiscent of another microtubule regulator, Apc2 [31]. We previously identified proteins required for Apc2 localization to branch points [27] and hypothesized that some of these may also target γTub-GFP. Reduction of mitochondria and actin regulators as well as Wnt signaling proteins disrupted Apc2-GFP localization [27]. RNAi lines that targeted proteins in the Wnt signaling group but not the other groups reduced γTub-GFP at dendrite branch points (Figs 1A–1C and S1B–S1E). Of Wnt signaling proteins, those broadly involved in multiple signaling pathways, including fz, dsh, CK1γ/gilgamesh (gish), and heterotrimeric G proteins [33–38], had phenotypes (Fig 1A and 1C). In addition, phenotypes occurred upon reduction of a subset of proteins specific to canonical Wnt signaling, including the scaffolding protein Axin [37] and arr, the *Drosophila* LRP5/6 ortholog [37, 39]. Knockdown of proteins specific to planar cell polarity (PCP) [38, 40] did not reduce γTub-GFP at branch points (Fig 1C). Surprisingly, canonical Wnt signaling proteins Apc and armadillo (arm) (β-catenin/arm) did not seem to play a role at branch points (Fig 1C). Because it is difficult to make firm conclusions from negative RNAi data, we examined expression of arm in the ddaE neuron using a GFP-tagged arm minigene (arm promoter driving full-length arm) that is fully functional and rescues a null arm mutant [41]. Arm-GFP was readily visible at borders of epithelial cells but was not detectable in neurons (S2A Fig). In contrast, a GFP insertion in the *gish* gene yielded visible fluorescence in neuronal nuclei and dendrites as well as epithelial cells (S2B Fig). Axin expression in ddaE neurons was confirmed using a validated [42] antibody (S2C Fig), and concentration of endogenous γTub at dendrite branch points was validated with a functional γTub-super-folder green fluorescent protein (sfGFP), which is tagged at the endogenous locus [43] (S2D Fig). Thus, although we confirmed expression of γTub, Axin, and gish in ddaE neurons, we could not detect expression or function of arm, the key transcriptional output of Wnt signaling, in these cells.

Although γTub plays a role in controlling microtubule nucleation and polarity in ddaE neurons, even strong reduction of function does not affect overall shape of these cells [26], perhaps because, under normal conditions, amplification of microtubule number through severing [44] can compensate for reduced nucleation. In larger ddaC neurons, dendrite simplification is seen when γTub is reduced [25]. The stronger effect on these large neurons likely represents increased demands on the cytoskeleton in the larger arbor that are less able to be compensated for by parallel pathways. We confirmed that, like γTub RNAi, RNAi hairpins targeting Wnt signaling proteins fz, dsh, and Axin had no effect on dendrite branching in ddaE neurons (S3 Fig). Similarly, no global changes in microtubule stability assessed by staining of acetylated tubulin were seen in fz, dsh, or Axin RNAi neurons (S4 Fig).

To confirm that a specific subset of canonical Wnt signaling proteins was required for γTub-GFP localization in dendrites, we used additional genetic approaches. Animals heterozygous for the hypomorphic *fz*^*F31*^ allele [45] had strongly reduced γTub-GFP at dendrite branch points (Fig 1E); the dotted line indicates the baseline signal expected from cytosolic GFP. Similar results were obtained with a small deficiency that removes the entire *fz2* gene and one neighboring gene (reptin [*rept*]) [46] and in animals heterozygous for two different strong loss-of-function *Axin* alleles, *Axin*^*18*^ [42] and *Axin*^*s044320*^ [47]. Combining Axin RNAi with a mutant allele (Axin A; *Axin*^*s044320*^/+) also reduced γTub-GFP at branch points to baseline. To confirm a role for Gαo, we overexpressed a dominant-negative guanosine diphosphate (GDP)-bound mutant [48], and this reduced γTub-GFP (Fig 1E). A constitutively active G protein alpha subunit s (Gαs)-guanosine triphosphate (GTP) [49] had no effect (Fig 1E). Finally, we used two shaggy (sgg) (glycogen synthase kinase 3 beta [GSK3β]) transgenes that contain mutations of a conserved regulatory phosphorylation site. The S9A version cannot be inhibited by phosphorylation and so is constitutively active, whereas the S9E version mimics the inactive phosphorylated form [50, 51]. The active mutant, but not the inactive mutant, reduced γTub-GFP localization to branch points (Fig 1E), suggesting an antagonistic role for sgg in the pathway. To test γTub-GFP localization in *fz* homozygotes (*fz*^*F31*^*/fz*^*P21*^, *fz*^*P21*^ is a strong loss-of-function allele [45, 52]), we used a different Gal4 driver that expresses in class I neurons (IG1-Gal4) and generated control data in a matched background. As expected, γTub-GFP was strongly reduced at branch points (Fig 1D and 1E). We conclude from this data that fz, fz2, arr, dsh, CK1γ, Gαo, Gαs, and Axin, as well as cnn and Plp, positively regulate γTub localization, whereas sgg may negatively regulate it (Fig 1B). We did not find any evidence that arm or PCP-specific proteins participate in localizing γTub.

### arr and fz act upstream of dsh, and dsh acts upstream of Axin, at dendrite branch points

**Fig 2:**
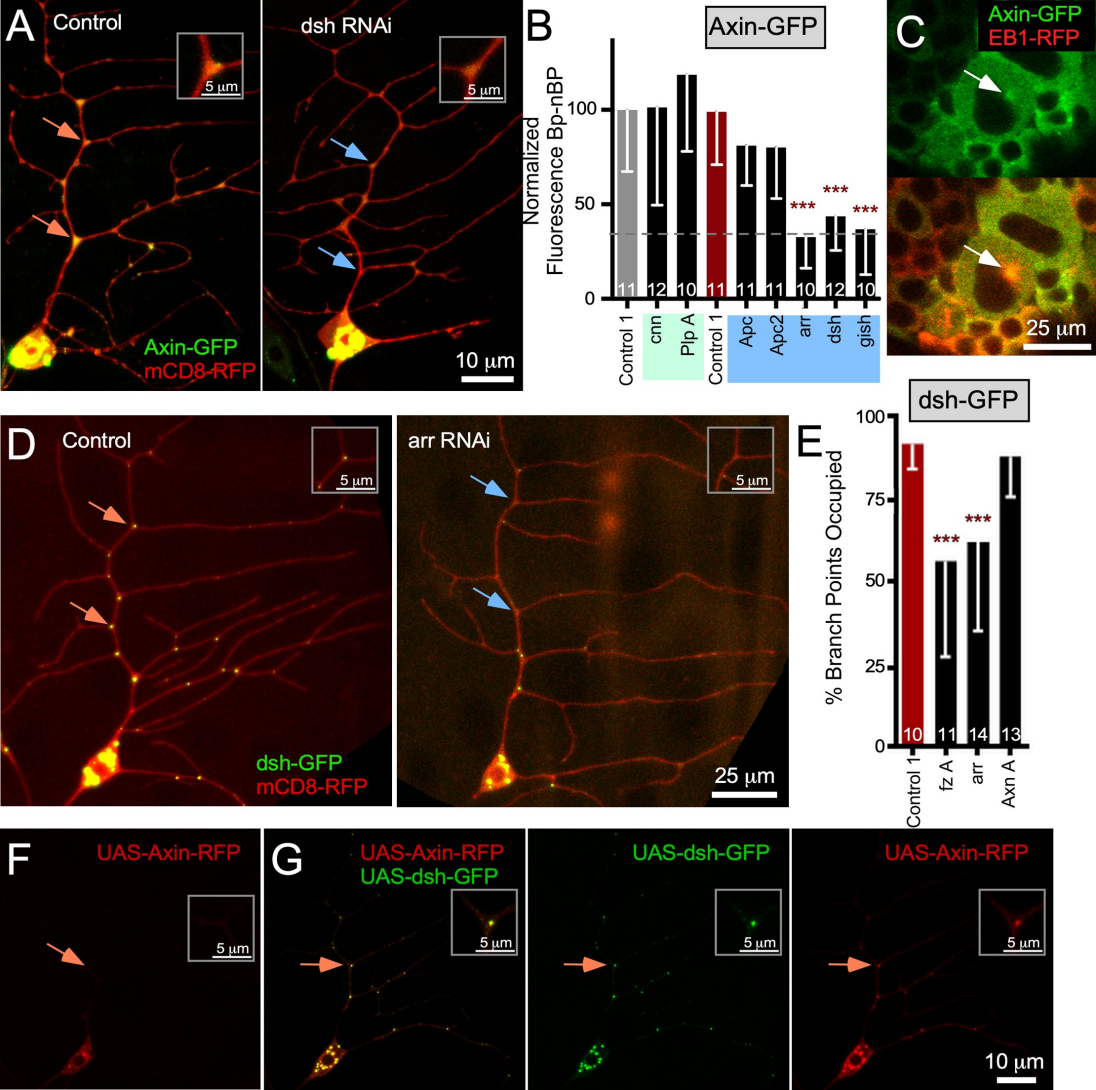
arr and fz act upstream of dsh, which is sufficient to recruit Axin to BPs. (A) Example images of ddaE neurons expressing UAS-Axin-GFP, UAS-mCD8-RFP, and either UAS-Rtnl2 RNAi (control 1) (VDRC 33320) or UAS-dsh RNAi (VDRC 101525) transgenes. Orange arrows indicate BPs with high Axin-GFP signal, and blue arrows indicate BPs with low signal. Insets in the top corner of each image indicate the top BP indicated in each image (B) Quantification Axin-GFP BP-nBP normalized fluorescence in ddaE neurons expressing different RNAi hairpins. (C) UAS-Axin-GFP and UAS-EB1-RFP were expressed using the pan neuronal driver elav Gal4. Live first instar larvae were mounted for imaging, and movies of neuroblasts in the brain were acquired (see S1 Movie). Still images from movies are shown. Centrosomes were identified as the site of EB1-GFP comet initiation and are identified with white arrows. (D) Example images of dsh-GFP and mCD8-RFP in neurons also expressing UAS-Rtnl2 RNAi (control 1) (VDRC 33320) and UAS-arr RNAi (BDSC 31473) transgenes. Orange arrows indicate BPs with high signal, and blue arrows indicate BPs with low signal; insets are as in other figures. (E) Quantification of dsh-GFP localization at BPs. BP occupancy was scored if there was a discrete dsh punctum present at each BP along the main branch of the comb dendrite. The number of cells (one per animal) is shown on the bars. Error bars indicate standard deviation. A linear regression was used to determine statistical significance. *p < 0.05, **p < 0.01, ***p < 0.001. (F) Representative image of UAS-Axin-RFP. (G) Panel of images showing UAS-Axin-RFP expressed with UAS-dsh-GFP using 221 Gal4. Refer to S1 Table for all genotypes and S1 Data for data used to generate graphs in (B) and (E). arr, arrow; Axn, Axin; BDSC, Bloomington Drosophila Stock Center; BP, branch point; dda, dorsal dendritic arborization; dsh, dishevelled; EB1, end-binding protein 1; fz, frizzled; GFP, green fluorescent protein; gish, gilgamesh; nBP, non–branch point; RFP, red fluorescent protein; RNAi, RNA interference; Rtnl2, reticulon 2; UAS, upstream activating sequence; VDRC, Vienna Drosophila Resource Center.

Consistent with a role in localizing γTub, a functional tagged Axin transgene [53]) concentrates at dendrite branch points (Fig 2A and [27]). We previously showed that fz, fz2, and Gαo are necessary for Axin-GFP branch point localization [27]. After finding that other Wnt signaling proteins act upstream of γTub localization, we wished to confirm that they also play a role in Axin targeting. Indeed, the same Wnt signaling proteins required for γTub positioning were required for Axin-GFP targeting. dsh, gish, and arr, but not Apc and Apc2, reduced Axin at branch points (Fig 2A and 2B). Unlike γTub, cnn and Plp RNAi did not affect Axin localization (Fig 2B). Similarly, cnn and Plp RNAi did not affect Apc2-GFP localization (S4 Fig), suggesting that these centrosomal proteins are specifically required upstream of γTub.

Although a role for Axin in dendritic localization of γTub was unexpected, there is some precedent for a relationship between Axin and γTub. In cultured mammalian cells and mouse oocytes, Axin localizes to the centrosome and is required for γTub recruitment [54, 55]. We therefore examined Axin localization in dividing *Drosophila* neuroblasts and found that Axin-GFP was concentrated at centrosomes marked with EB1-RFP (Fig 2C and S1 Movie).

We next tested whether dsh, the other Wnt signaling scaffold involved in γTub-GFP localization, had a similar distribution to Axin. Dsh-GFP under UAS control localized to puncta present at over 90% of branch points (Fig 2D and 2E). Axin-GFP also clustered in puncta, although a more diffuse background was also seen with this marker (Fig 2A). To rule out that the dsh localization pattern was due to overexpression, we examined dsh-Clover, a derivative of an enhanced green fluorescent protein (EGFP)-tagged functional transgene expressed using its own regulatory sequences [56] tagged with the brighter Clover [57] fluorescent protein (S6 Fig). dsh-Clover also localized to puncta in neurons (S6B Fig). Because the UAS version was easier to image, we generated a tester line with this transgene. Although RNAi targeting Axin did not reduce dsh-GFP at branch points, the reduction of the membrane proteins fz and arr did (Fig 2D and 2E). These data suggest that dsh acts downstream of fz and arr but upstream of Axin at branch points.

To further confirm that dsh is upstream of Axin in dendrites, we used an RFP-tagged Axin transgene that is largely diffuse on its own (Fig 2F). When expressed with dsh-GFP, Axin-RFP was recruited to puncta at dendrite branch points and in the cell body that were labeled with GFP (Fig 2G). These data indicate that, in dendrites, Wnt receptors fz and arr act upstream of dsh, which in turn acts upstream of Axin.

### Wnt signaling proteins are required for normal microtubule polarity in dendrites

**Fig 3:**
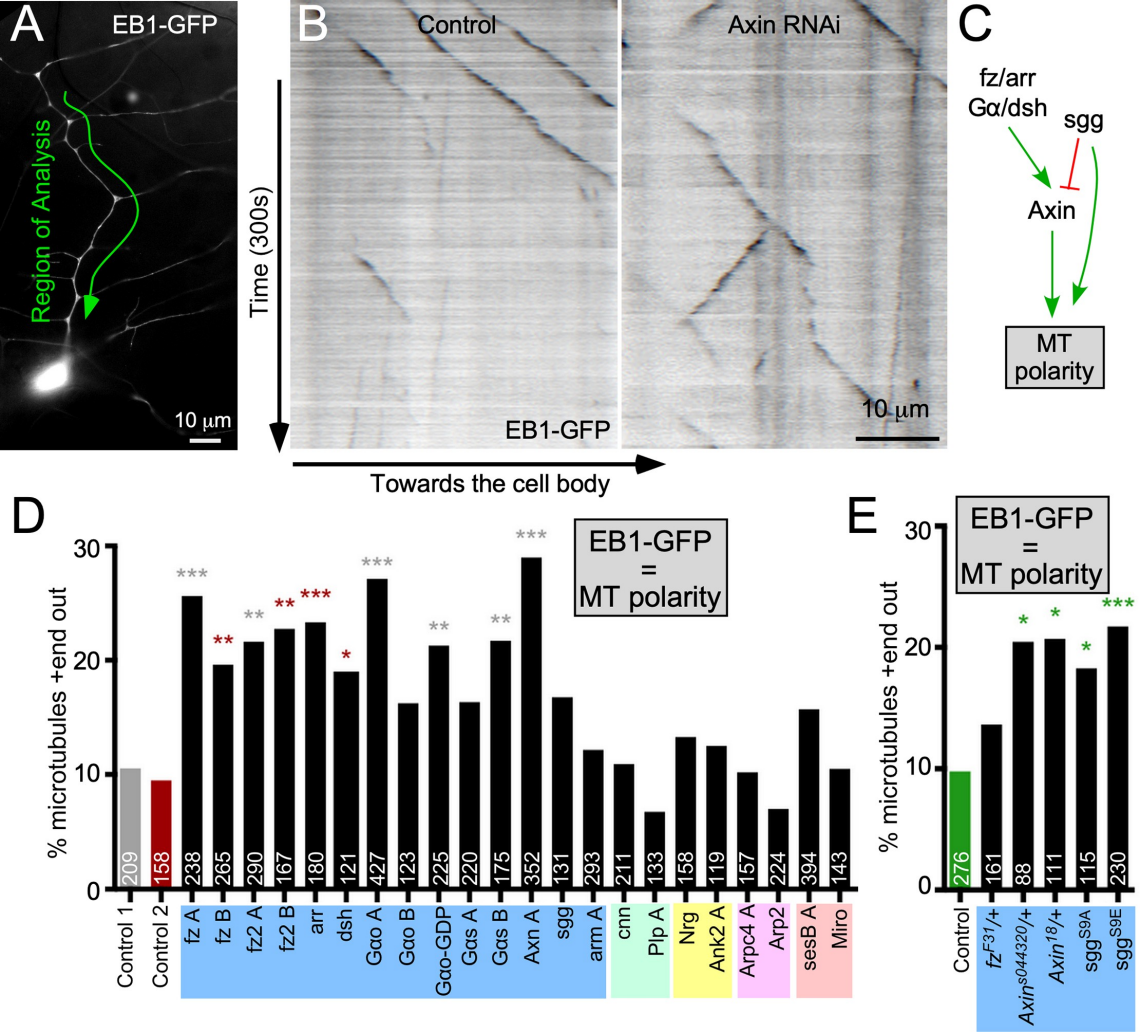
Wnt signaling proteins are required for minus-end-out microtubule polarity in dendrites. (A) An overview of the ddaE neuron dendrite arbor is shown. The main trunk of the ddaE neuron used for EB1-GFP movies is highlighted. (B) The 300-second movies of EB1-GFP were acquired in the ddaE dorsal dendrites, and kymographs were generated in Fiji. Cells also expressed either UAS-Rtnl2 RNAi (control 1) (VDRC 33320) (left) or UAS-Axin RNAi (VDRC 7748) (right). (C) A summary of data in earlier figures as well as some previous data [27] is shown. (D) Quantification of EB1-GFP comet direction in the main trunk of the ddaE dendrite in animals expressing hairpin RNAi’s. The percentage of microtubules oriented plus-end-out is plotted as a summed value across all cells for each genotype. The numbers on each bar are total EB1-GFP comets counted, and at least 15 cells were analyzed for each genotype, with one cell per animal. Data in panel D were collected by two different individuals; the gray bar shows control data from one individual, and the red bar is from the other individual. Experimental genotypes analyzed by an individual were compared with their own control data, and significance stars are color-coded to indicate the comparisons. (E) Quantification of microtubule polarity from mutant and dominant-negative strains compared with control data without an RNAi transgene. A logistic regression was used to determine significance. *p < 0.05, **p < 0.01, ***p < 0.001. Refer to S1 Table for all genotypes and S1 Data for data used to generate graphs in (D) and (E). Ank2, Ankyrin 2; Arp2, actin related protein 2; Arpc4; actin related protein c4; arr, arrow; Axn, Axin; dda, dorsal dendritic arborization; EB1, end-binding protein 1; Gα, G protein alpha subunit; GDP, guanosine diphosphate; GFP, green fluorescent protein; Miro, mitochondrial rho; MT, microtubule; Nrg, Neuroglian; Plp, Pericentrin-like protein; RNAi, RNA interference; Rtnl2, reticulon 2; sgg, shaggy; UAS, upstream activating sequence; VDRC, Vienna Drosophila Resource Center.

So far, we have used γTub-GFP as a proxy for nucleation sites. As a first functional test of a role for Wnt signaling proteins in controlling dendritic nucleation, we took advantage of the polarity phenotype generated by loss of γTub. A strong reduction of γTub23C (the somatic γTub) generates mixed polarity in ddaE dendrites, shifting the percentage of plus-end-out microtubules from about 10 to 25 [26]. We hypothesized that reduction of γTub at branch points would phenocopy the *γTub* phenotype and disrupt dendrite microtubule polarity. We used the direction of EB1-GFP comet movement [58] as a readout of microtubule polarity. As expected, in control neurons, about 10% of microtubules were plus-end-out (Fig 3B and 3D). In many genetic backgrounds in which γTub-GFP was reduced at branch points, polarity was more mixed (Fig 3 and S2 Movie) and was comparable to that in *γTub*^*A14-9*^*/γTub*^*A15-2*^ mutant animals [26]. In contrast with other assays, inactive sgg had a phenotype here, suggesting that sgg may have a positive function in maintaining dendritic microtubule polarity not necessarily related to nucleation (Fig 3C and 3E). Not all of the genetic backgrounds that reduced γTub-GFP localization resulted in changes in dendrite microtubule polarity. For example, the *fz* heterozygous mutant animals had reduced γTub-GFP at dendrite branch points (Fig 1E) but had normal microtubule polarity, as did cnn and plp RNAi (Fig 3D and 3E). One explanation consistent with our previous results is that γTub function must be strongly reduced to affect microtubule polarity [26, 29]. We therefore wished to use a more sensitive functional assay to further test the requirement of candidate proteins in nucleation.

### Wnt signaling proteins are required to increase microtubule dynamics in response to axon injury

Axon severing results in increased microtubule dynamics (number of growing plus ends) in parts of the neuron remaining connected to the cell body in *Drosophila* [59] and mammals [60]. This increase in dynamics is dependent on microtubule nucleation [29]. Unlike polarity in uninjured neurons, increased microtubule dynamics after axon injury is affected by loss of one copy of *γTub23C* or by RNAi targeting γTub [29] and so is more sensitive to partial reduction of nucleation.

**Fig 4:**
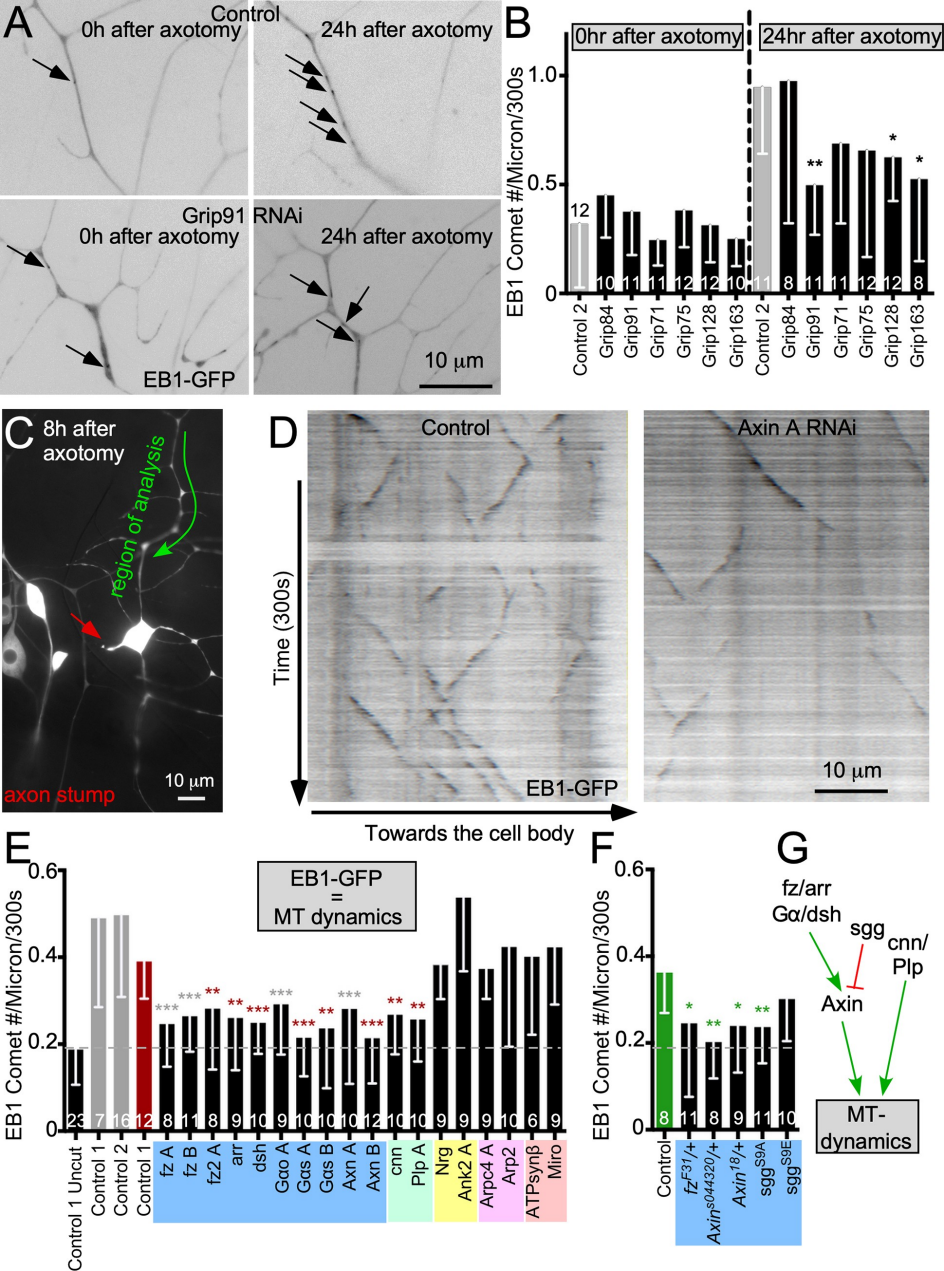
Wnt signaling proteins are required for microtubule dynamics induced by axon injury. (A) UAS-EB1-GFP was expressed in ddaE neurons with different RNAi hairpins. Axons were severed with a pulsed UV laser, and 400-second (1 frame per 2 seconds) movies were acquired immediately after injury and 24 hours after injury in the dendrite. These values were normalized to 300 seconds so as to more accurately compare them with data shown in (E) and (F). However, the quantification in (E) and (F) differs slightly than this set because of the set 10-μm length for each video. In (E) and (F), there is a variable 20- to 30-μm distance. With a set short distance of 10 μm, the probability of more comets passing through in the same given time is increased because of EB1 sustained run length. Individual frames from the movies are shown. (B) Quantification of EB1-GFP comets per micrometer and 300 seconds for all knockdowns immediately following injury and 24 hours after injury are shown in different RNAi backgrounds. The dashed line separates 0-hour from 24-hour measurements. The number of cells analyzed is noted on each bar. A linear regression was used to determine statistical significance. *p < 0.05, **p < 0.01, ***p < 0.001. (C) An overview of a ddaE neuron 8 hours after axon injury is shown. The axon stump is indicated with a red arrow. (D) Fiji-generated kymographs depicting microtubule dynamics 8 hours postaxotomy in UAS-Rtnl2 RNAi (control 1) (VDRC 33320) (left) and UAS-Axin RNAi (VDRC 7748) (right) are shown. (E) Quantification of microtubule dynamics (comet number per micrometer and 300 seconds) in animals in different knockdown conditions is shown. A dashed line indicates baseline, uninjured, control microtubule dynamics. This dashed line will continue throughout the figures that show microtubule dynamics. Gray and red control data were generated by two different individuals, and experimental data were compared with the control by the same individual as indicated by star color. (F) Quantification of microtubule dynamics in non-RNAi genetic backgrounds is shown. Error bars indicate standard deviation. A linear regression was used to determine statistical significance. *p < 0.05, **p < 0.01, ***p < 0.001. (G) A schematic summarizing the data in panels E and F is shown. Refer to S1 Table for all genotypes and S1 Data for data used to generate graphs in (B), (E), and (F). Ank2, Ankyrin 2; Arpc4, actin related protein c4; arr, arrow; ATPsynβ, ATP Synthase β; Axn, Axin; cnn, centrosomin; dda, dorsal dendritic arborization; dsh, dishevelled; EB1, end-binding protein 1; fz, frizzled; GFP, green fluorescent protein; Miro, mitochondrial rho; MT, microtubule; Nrg, Neuroglian; Plp, Pericentrin-like protein; RNAi, RNA interference; Rtnl2, reticulon 2; sgg, shaggy; UAS, upstream activating sequence; VDRC, Vienna Drosophila Resource Center.

Before using this as a nucleation assay, we wished to confirm that the injury response relies on classical microtubule nucleation through the γTub ring complex (γTuRC). The γTuRC contains gamma tubulin ring protein (Grip) 91 and 84, which together make the γTub small complex (γTuSC), as well as four additional subunits, Grip71, Grip75, Grip128, and Grip163, which bring together γTuSCs to form the γTuRC. We used RNAi to reduce γTuRC proteins in neurons and assayed microtubule dynamics in dendrites immediately after axon injury and 24 hours later. Right after injury, microtubule plus-end number was similar in all genetic backgrounds (Fig 4A and 4B). At 24 hours after injury, when the number of growing plus ends in control dendrites was more than 2-fold elevated compared with uninjured neurons (Fig 4A and 4B), neurons expressing RNAi hairpins targeting some of the γTuRC components did not increase microtubule dynamics to the same extent (Fig 4A and 4B). The Grip84 RNAi line did not reduce dynamics in response to injury and may be ineffective. We conclude that the injury-induced increase in microtubule dynamics in dendrites depends on γTuRC and is thus a functional assay for classic microtubule nucleation.

To test whether reduction of γTub-GFP in dendrites predicts reduced ability to nucleate microtubules in response to stress, we assayed microtubule dynamics in the ddaE comb dendrite 8 hours after axon injury in different genetic backgrounds (Fig 4C and S3 Movie). Consistent with γTub localization results (S1 Fig) and the microtubule polarity assay (Fig 3D), disruption of branched actin (actin related protein c4 [Arpc4], Arp2 RNAi), mitochondria (mitochondrial rho [Miro], ATP Synthase β [ATPsynβ] RNAi), or Neuroglian (Nrg)/Ankyrin 2 (Ank2) did not block the increase in microtubule dynamics after injury (Fig 4E). However, reducing any of the proteins that were required for γTub localization to dendrite branch points reduced microtubule dynamics in dendrites after axon injury (Fig 4D, 4E and 4F). Microtubule polarity changes were also observed in response to injury (Fig 4D), as expected [59]. These changes are not dependent on microtubule nucleation [29] and so were not tracked in this assay.

As predicted, injury-induced nucleation was more sensitive to reduction in proteins that target γTub than the polarity assay. We conclude not only that fz/arr/dsh/Gα/Axin, cnn, and Plp are required to position γTub at dendrite branch points but also that disruption of γTub localization has functional consequences for microtubule nucleation in dendrites (Fig 4G).

### Axin and dsh localize to Rab5 endosomes in dendrites

**Fig 5:**
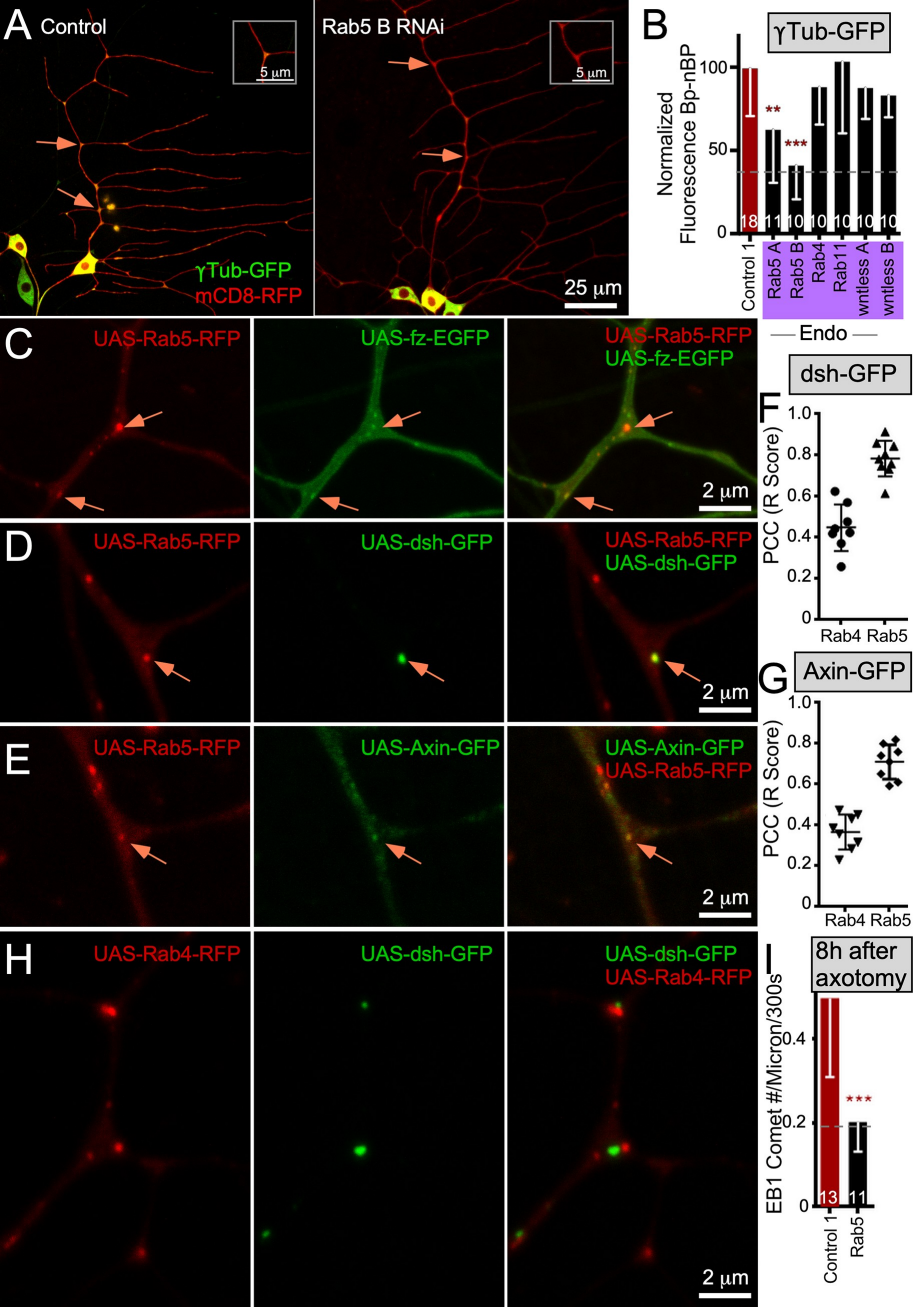
Wnt signaling proteins localize to Rab5 endosomes. (A) Example images of UAS-γTub-GFP localization in ddaE neurons expressing UAS-Rtnl2 RNAi (control 1) (VDRC 33320) and UAS-Rab5 RNAi (VDRC 34096) hairpins. Membranes were marked with UAS-mCD8-RFP to see cell shape. Orange arrows indicate BPs with high γTub-GFP signal, and blue arrows indicate BPs with low γTub-GFP signal. Insets in the top corner of each image show the top BPs highlighted with an arrow in each image. (B) Quantification of γTub-GFP at BPs is shown in larvae expressing different RNAi hairpins targeting endosomal proteins or a wnt secretion protein wntless. Values were generated by subtracting mean nBP fluorescence from BP fluorescence for each cell; normalized fluorescence values are shown. Sample sizes are shown within the bars. Error bars indicate standard deviation. A linear regression was used to determine statistical significance. *p < 0.05, **p < 0.01, ***p < 0.001. (C–E) Example colocalization image of UAS-Rab5-RFP coexpressed with UAS-fz-eGFP, UAS-dsh-GFP, or UAS-Axin-GFP. The orange arrows point to puncta of colocalization between the two markers in each case. For all colocalization experiments, sequential scanning was used to ensure no bleed through between the markers. (F and G) Plot of PCC between UAS-Rab4-RFP or UAS-Rab5-RFP with either UAS-dsh-GFP or UAS-Axin-GFP. The y-axis indicates the R score, with 1 being positive correlation, 0 meaning no correlation, and −1 meaning negative correlation. (H) Examples images of UAS-Rab4-RFP coexpressed with UAS-dsh-GFP. (I) Quantification of microtubule dynamics (comet number per micrometer and 300 seconds) following laser axotomy in control animals or animals expressing UAS-Rab5 RNAi (VDRC 34096). Error bars indicate standard deviation. A linear regression was used to determine statistical significance. *p < 0.05, **p < 0.01, ***p < 0.001. Refer to S1 Table for all genotypes and S1 Data for data used to generate graphs in (B), (F), (G), and (I). γTub, γTubulin; BP, branch point; dda, dorsal dendritic arborization; dsh, dishevelled; EB1, end-binding protein 1; eGFP, enhanced green fluorescent protein; fz, frizzled; GFP, green fluorescent protein; nBP, non–branch point; PCC, Pearson’s correlation coefficient; RFP, red fluorescent protein; RNAi, RNA interference; Rtnl2, reticulon 2; UAS, upstream activating sequence; VDRC, Vienna Drosophila Resource Center.

The involvement of membrane proteins in γTub localization suggested that either the plasma membrane or an organelle might be used as a platform to organize nucleation sites. We examined Golgi and endosome markers in ddaE neurons and found that endosomes localized to most branch points, whereas clear spots of Golgi were only seen occasionally in proximal branch points (S7A Fig). RNAi transgenes targeting lava lamp (lva), a protein required for Golgi transport into *Drosophila* dendrites [24], chromosome bows (chb), the *Drosophila* cytoplasmic linker associated protein (CLASP; CLASPs in mammalian cells help microtubules grow from the Golgi [61]), and cytoplasmic linker protein-190 kDa (CLIP-190), which is a binding partner of lva [62], did not alter γTub-GFP at branch points (S7B Fig). In contrast, Rab5 RNAi, but not knockdown of other Rabs, reduced γTub-GFP localization (Fig 5A and 5B). Endocytosis is required for efficient Wnt secretion and involves the cargo chaperone wntless [63]. However, wntless RNAi did not have a phenotype (Fig 5B), suggesting that the Rab5 phenotype does not occur because of reduced ligand generated by the neuron.

To determine whether Wnt signaling proteins localize to dendritic endosomes, we coexpressed tagged fz, Axin, and dsh with Rab4, Rab5, and Rab11. Unlike tagged Axin and dsh, fz-EGFP was observed throughout the plasma membrane as well as defined puncta at branch points (Fig 5C). Fz, dsh, and Axin puncta colocalized with Rab5 but not Rab4 or 11 (Figs 5C–5H and S7D). In the cell body, expression levels of most markers were very high, but Axin formed large puncta that overlapped with a subset of Rab5-labeled structures (S8A Fig).

To make sure that colocalization was not due to overexpression of tagged transgenes, we used mCherry-Rab5 [64] and dsh-Clover, which were controlled by their own regulatory sequences (S6 Fig). A subset of mCherry-Rab5 puncta aligned with dsh-GFP expressed in neurons (S6A Fig). In neurons, dsh-Clover puncta aligned with mCherry-Rab5 puncta (S6B Fig). A functional role for Rab5 in control of nucleation was supported by failure to up-regulate microtubule dynamics after axon injury in Rab5 RNAi neurons (Fig 5I).

To try to understand why the Golgi complex rather than endosomes was previously implicated in dendritic nucleation [25, 32], we examined tagged versions of mannosidase II (ManII), the major Golgi marker used in the previous studies. Different tagged ManII transgenes were present in zero to one large puncta in dendrites (S7A Fig), as well as many smaller spots that could be seen with higher laser power (S7E Fig). Some of these smaller spots colocalized with Rab5-GFP (S7E Fig), suggesting leakage of markers between organelles. To make sure that it was not the endosomal marker leaking into Golgi, we used a fly line that has the start codon of the *Rab5* gene replaced with the enhanced yellow fluorescent protein (EYFP) coding sequence [65]. ManII-RFP was seen in puncta labeled with endogenous EYFP-Rab5 (S7E Fig), indicating that ManII, not Rab5, is mislocalized in these cells. Therefore, it is possible that the structures assumed to be Golgi outposts in other studies were actually early endosomes into which overexpressed ManII had leaked. In summary, the data suggest that Wnt signaling proteins can be found on a subset of early endosomes in dendrites.

### New growing plus ends can initiate at early endosomes in dendrites

**Fig 6:**
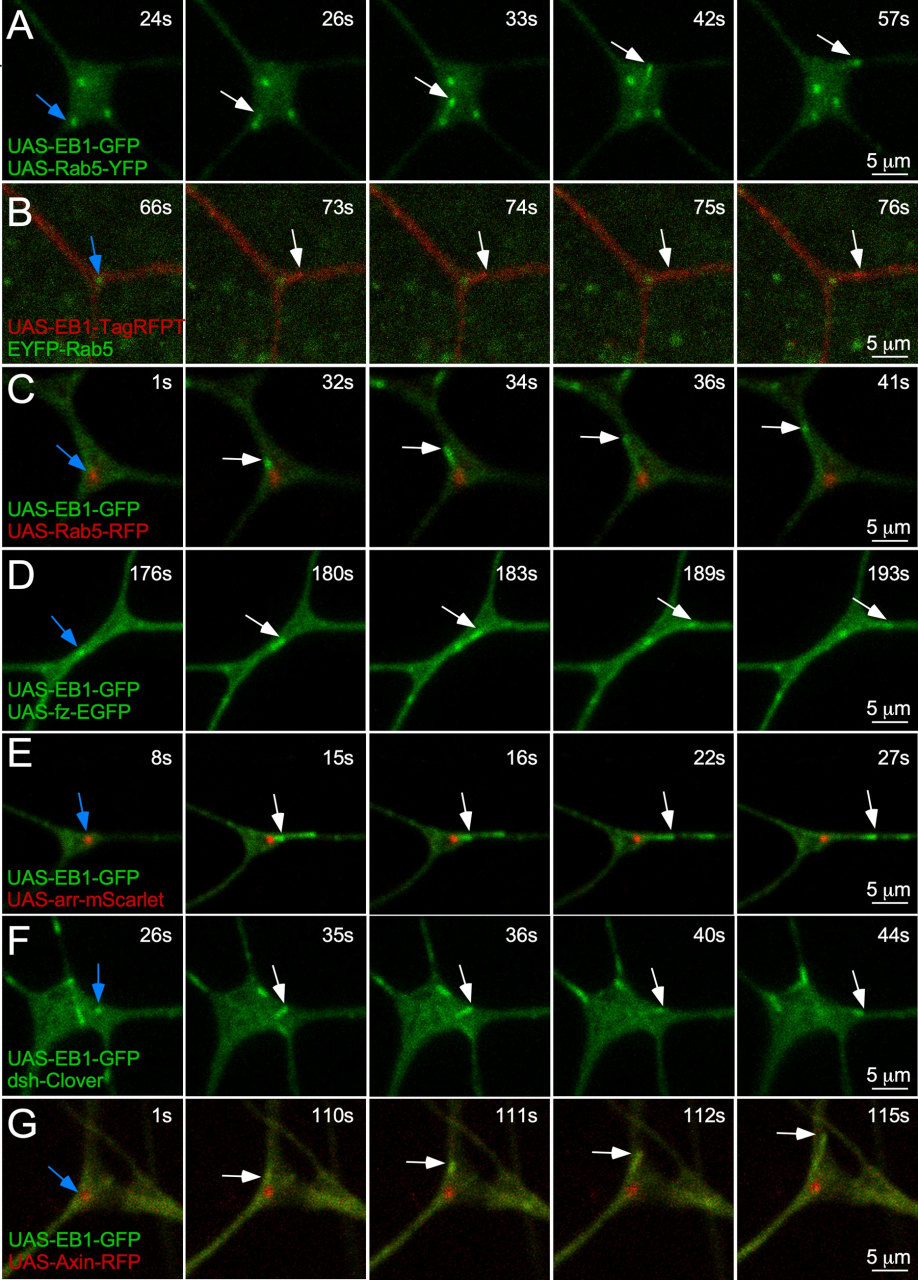
Microtubules can initiate growth from endosomes in dendrites. (A–G) Example five-frame stills of microtubule comet formation off either Rab5 endosomes or wnt proteins. UAS-Rab5-GFP or UAS-Rab5-RFP are shown coexpressed with UAS-EB1-GFP. In addition, UAS-EB1-TagRFPT is shown expressed with endogenous EYFP-Rab5. UAS-fz-eGFP is shown coexpressed with UAS-EB1-TagRFPT. Finally, UAS-arr-RFP, endogenous dsh-Clover, and UAS-Axin-RFP are shown with UAS-EB1-GFP. In all examples, the first frame includes a blue arrow to show the endosome or wnt proteins off of which the microtubule comet will initiate. Subsequent frames track movement of the microtubule with a white arrow. The time stamp at the top-right corner correlates to the time point in the corresponding S4–S7, S10 and S11 Movies. arr, arrow; dsh, dishevelled; EB1, end-binding protein 1; eGFP, enhanced green fluorescent protein; EYFP, enhanced yellow fluorescent protein; fz, frizzled; GFP, green fluorescent protein; RFP, red fluorescent protein; UAS, upstream activating sequence.

The finding that dsh and Axin localized to endosomes in dendrites implicated these as potential sites of nucleation. We used formation of new comets by EB1-GFP as a readout of nucleation. Whereas new comets can be a result of catastrophe rescue, within branch points, comet formation has been linked to nucleation [26]. In movies of tagged protein pairs, we captured EB1 comets initiating from puncta labeled with Rab5 as well as each of the Wnt signaling markers that colocalized with Rab5 (Figs 6 and S9 and S4–S12 Movies). In addition to UAS-driven transgenes, Rab5 and dsh under endogenous control were seen as sites of comet initiation (Fig 6B and 6F and S5 and S9 Movies). EYFP-Rab5 has the fluorescent protein coding sequence inserted at the start codon of the genomic Rab5 [65]. In some of the movies, EB1 was in the same channel as the other marker, but it was easy to distinguish endosomes and microtubule plus ends based on their behavior. In some examples, including that in Fig 6A and S4 Movie, the endosome was pulled along by the newly growing microtubule, providing strong support for the association of the microtubule with the endosome. These events are consistent with recruitment of nucleation machinery to Rab5 endosomes by Wnt signaling proteins.

**Fig 7:**
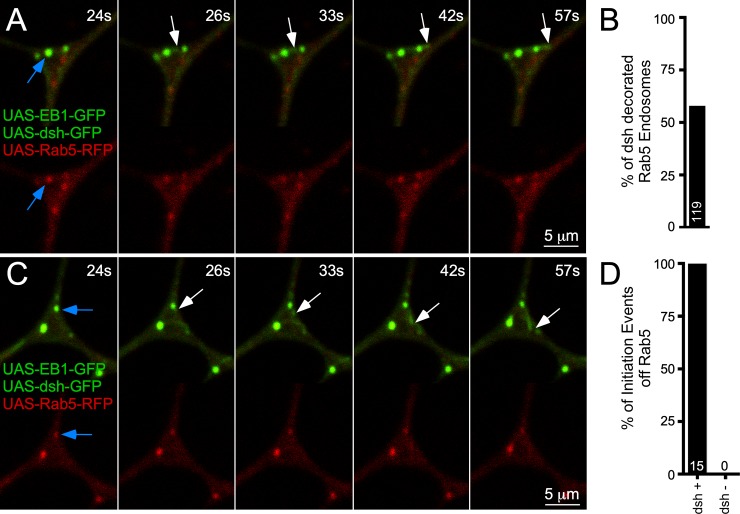
Microtubules initiate from dsh-decorated early endosomes. (A and C) Frames from two representative videos of a class I neuron expressing UAS-EB1-GFP, UAS-dsh-GFP, and UAS-Rab5-RFP. Top panels are the merged image, and bottom is the UAS-Rab5-RFP. A blue arrow is used to show the endosome from which an EB1 comet will initiate in subsequent frames, indicated with white arrows to track movement. The time stamp at the top-right corner correlates to the time point in the corresponding S13 and S14 Movies. (B) Quantification of the percentage of Rab5 endosomes that are labeled with UAS-dsh-GFP. Sample size is shown in the bar and indicates total number of Rab5-RFP puncta that were counted in branch points during 25 300-second videos. (D) Quantification of EB1 comet events that initiate from dsh-positive or dsh-negative Rab5 endosomes during the same 300-second videos. Sample size, which represents total comet number, is shown in the bar. Refer to S1 Table for all genotypes and S1 Data for data used to generate graphs in (B) and (D). dsh, dishevelled; EB1, end-binding protein 1; GFP, green fluorescent protein; RFP, red fluorescent protein; UAS, upstream activating sequence.

To further test whether a specific type of signaling endosome is involved in dendritic microtubule nucleation, we coexpressed EB1-GFP, dsh-GFP, and Rab5-RFP. We counted dsh and Rab5 puncta in branch points of 25 cells and found that all dsh puncta overlapped with Rab5 puncta but that some Rab5 puncta did not contain dsh signal. When we quantitated the overlap, we found that 60% of Rab5 puncta colocalized with dsh (Fig 7A and 7B). Thus, dsh is found at a subset of endosomal structures marked with Rab5. Based on the involvement of dsh in γTub localization, we hypothesized that only Rab5 structures with dsh should be sites of comet initiation. Indeed, when we examined 5-minute movies of dendrite arbors from 25 cells, we identified 15 comets that initiated at Rab5-labeled puncta, and all of these also contained dsh signal (Fig 7 and S13 and S14 Movies). We conclude that new microtubules can initiate at a subset of endosomes that contain Wnt signaling proteins.

### Axin is sufficient to localize γTub to ectopic cellular sites

**Fig 8:**
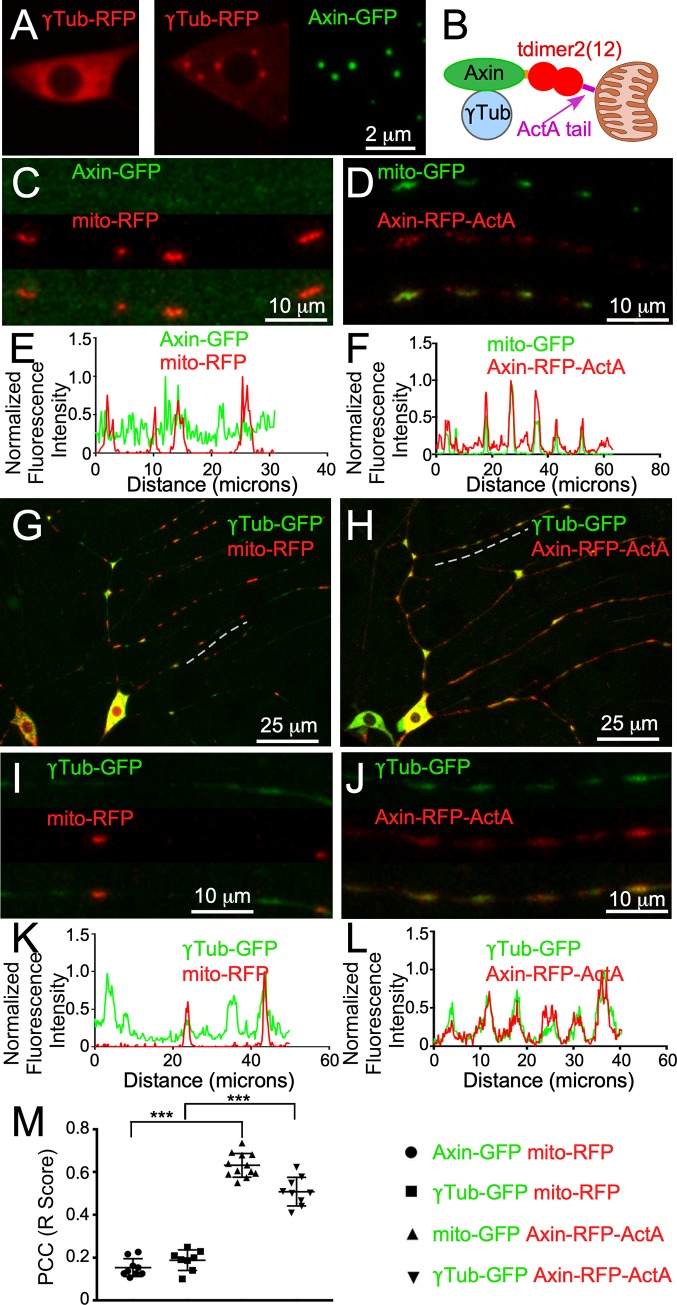
Axin is sufficient to localize γTub to ectopic cellular sites. (A) Images show the localization of UAS-γTub-RFP in the cell body of a ddaE neuron when expressed alone (left) or when coexpressed with UAS-Axin-GFP (right) using the 221-Gal4 driver. (B) A diagram of the chimeric protein used to tag Axin with RFP (tdimer2[12]) and target it to mitochondria is shown. (C and D) Example images of enlarged regions within the secondary ddaE dendrites in cells expressing a mitochondrial marker and either UAS-Axin-GFP or UAS-Axin-RFP-ActA. (E and F) Fluorescence intensity measurements from line traces of the dendrite regions shown in (B) and (C). (G and H) UAS-γTub-GFP was coexpressed with either UAS-mito-RFP or UAS-Axin-RFP-ActA using 221Gal4. Overview images of the entire ddaE dendrite arbor are shown. (I and J) Enlarged images of the regions within the secondary ddaE dendrites, indicated by the dashed lines in (G) and (H). (K and L) Normalized fluorescence measurements from line tracings of the regions in (G) and (H). (M) A plot of the PCC for the four conditions (see the key to the right of the graph). The y-axis indicates the R score, with 1 being a positive correlation, 0 meaning no correlation, and −1 indicating a negative correlation. Refer to S1 Table for all genotypes and S1 Data for data used to generate graphs in (E), (F), (K), (L), and (M). γTub, γTubulin; ActA, actin assembly promoting protein A; dda, dorsal dendritic arborization; GFP, green fluorescent protein; Mito, mitochondrial; PCC, Pearson’s correlation coefficient; RFP, red fluorescent protein; UAS, upstream activating sequence.

Although we observed localization of fz, dsh, and Axin to endosomes, we were not normally able to observe distinct puncta of γTub, even with endogenously tagged γTub (S2D Fig) and antibody staining of γTub [26]. One exception to diffuse γTub was when we overexpressed a red version with tagged Axin. By itself, γTub-RFP was diffuse in the cell body (Fig 8A), but when paired with Axin-GFP, it was recruited to very defined puncta in the cell body (Fig 8A) and also in dendrites (S8B Fig). We therefore hypothesized that Axin might be sufficient to recruit γTub to specific intracellular locations, but at branch points, this was normally too transient to detect above diffuse background. To test whether Axin was sufficient to localize γTub, we used a short sequence from the actin assembly promoting protein A (ActA) protein of *Listeria monocytogenes* that targets the outer mitochondrial membrane in mammalian [66] and *Drosophila* [67] cells to generate an Axin fusion protein that we predicted would be targeted to mitochondria (Fig 8B). When expressed in ddaE neurons with mitochondrial (mito)-GFP, Axin-RFP-ActA colocalized with mitochondria; this pattern was particularly noticeable in the linear tips of the dorsal comb dendrite (Fig 8D and 8F). In contrast, Axin-GFP was present at very low levels in this region and did not align with mitochondria (Fig 8C and 8E). Because we were able to target Axin to mitochondria, we asked whether γTub-GFP would be concentrated around mitochondria. In the absence of mitochondrial Axin, γTub-GFP fluorescence was not strongly correlated with mitochondria (Fig 8G, 8I and 8K). However, when mitochondria were coated with Axin-RFP-ActA, γTub-GFP fluorescence much more closely followed the pattern of RFP fluorescence such that all γTub-GFP peaks in the regions analyzed were associated with RFP peaks (Fig 8H, 8J and 8L). To more quantitatively assess colocalization across multiple cells, we generated Pearson’s correlation coefficients from the comb dendrite for each set of markers (Fig 8M). This analysis was consistent with line tracings. From this data, we conclude that Axin is sufficient to recruit γTub to specific sites in *Drosophila* neurons.

In addition to Wnt signaling proteins, cnn is required to position γTub at branch points (Fig 1C) and seems to act in parallel to or downstream of Axin (Fig 2B). If it acts with γTub downstream of Axin, we hypothesized that Axin-RFP-ActA might also recruit cnn-GFP to mitochondria. Indeed, when we paired these two markers, we found that cnn-GFP could be relocalized to mitochondria in the same way that γTub was (S10 Fig).

Because cnn can activate microtubule nucleation [68, 69], we hypothesized that ectopic γTub and cnn on mitochondria might convert mitochondria into nucleation sites. We therefore combined Axin-RFP-ActA with EB1-GFP to determine whether comets would initiate at mitochondria. In control neurons, more comet initiation, or spawning events, occurred at branch points compared with between branch points (S11A and S11B Fig). When one copy of an *Axin* null allele was introduced into the background, spawning at branch points was reduced (S11A and S11B Fig). Neurons expressing mito-RFP had a similar pattern of spawning to neurons expressing only EB1-GFP (S11C and S11D Fig), whereas ectopic mitochondrial Axin increased spawning at branch points and between them (S11C and S11D Fig). The increase in comet initiation both at branch points and between them is consistent with localization of mitochondria to 80% of branch points [27] as well as to intervening regions (Fig 8G). On rare occasions, we saw multiple spawning events initiating from the region within a dendrite in which Axin-RFP-ActA was concentrated (S15 Movie). Axin-GFP expression without mislocalization to mitochondria did not alter microtubule dynamics in dendrites (S11E–S11G Fig). We conclude that Axin is necessary for normal dendritic microtubule dynamics and sufficient to increase microtubule dynamics when ectopically expressed.

## Discussion

It was particularly intriguing to find integral membrane signaling proteins required for noncentrosomal microtubule nucleation. Although Wnt signaling has been linked to microtubule plus-end regulation in axon growth cones [70] and regulation of microtubule stability and spindle orientation [71], the only connection to the minus end is localization of some cytoplasmic Wnt signaling proteins like Axin to the centrosome in dividing cells [54, 72]. Here, we demonstrate that a Wnt signaling pathway acts upstream of microtubule nucleation in a postmitotic cell. Not only were many canonical Wnt signaling proteins required for γTub-GFP to accumulate at branch points, but Axin and dsh themselves concentrated at branch points. In addition, the scaffolding protein Axin was able to recruit γTub-GFP and the nucleation activator cnn to mitochondria when tethered to them. Moreover, reduction of Wnt signaling proteins phenocopied loss of γTub in two functional nucleation assays, indicating that most or all dendritic nucleation occurs downstream of this pathway. Although this pathway seems to be the major regulator of dendritic nucleation, neurons are quite resilient to its loss under baseline conditions, and the simple ddaE neurons have normal arbor shape. This is likely because parallel pathways can be used to generate new minus ends. For example, microtubule severing can be used to generate new plus and minus ends and amplify microtubule number [44, 73]. In many cell types, minus ends generated when a microtubule is severed are recognized by minus-end binding proteins in the calmodulin-regulated spectrin-associated protein (CAMSAP)/Patronin family [74, 75]. In *C*. *elegans*, γTub-mediated microtubule nucleation has been shown to act in parallel and quite redundantly with Patronin to regulate microtubule organization [76]. We have recently shown that Patronin-mediated minus-end growth is an important regulator of dendritic microtubules in *Drosophila* [77], so it is possible that microtubule severing in conjunction with Patronin recruitment to minus ends can compensate for nucleation under most normal circumstances. Consistent with this hypothesis, phenotypes from reduction of nucleation or Patronin become more evident after severe stress, including axon [29] or dendrite [77, 98] injury.

Although we consistently found that partial loss of function (RNAi or heterozygous mutants) for fz, fz2, arr, dsh, Gao, Gas, and Axin reduced γTub localization and/or function, we could not find any evidence that β-catenin/arm, the key transcription factor that is the output of canonical Wnt signaling, was involved. In addition, an arm protein trap showed clear expression in epidermal cells but was not seen in dendritic arborization neurons (da neurons). Because Axin itself was sufficient to recruit γTub, there was no strong rationale for a transcriptional regulator to mediate signaling between fz/arr and microtubule nucleation. We propose that canonical Wnt signaling proteins are co-opted in dendrites to directly recruit nucleation complexes to endosomes. Because this is a variant of canonical Wnt signaling that unexpectedly seems not to involve β-catenin, we term this pathway apocryphal Wnt signaling in reference to the Apocrypha, ancient writings found in only some versions of the Bible.

The involvement of arr as well as dsh and Axin suggests that a signalosome might be involved in dendritic Wnt signaling. Signalosomes form when wnt ligands bind to fz and LRP5/6 at the plasma membrane, triggering recruitment and multimerization of dsh and Axin [78]. The normal output of signalosome formation is release of β-catenin from the destruction complex and its subsequent stabilization and transit to the nucleus to activate transcription [78]. Signalosomes assemble at the plasma membrane [79, 80]. Endocytosis generally seems to promote Wnt signaling [63], although in many contexts the signalosome itself is disassembled upon endocytosis [63, 80]. It is not clear whether signalosomes persist after endocytosis, though in some *Drosophila* cells, dsh and arr are localized to endosomes [28]. In dendrites, puncta of fz, dsh, and Axin colocalized with Rab5 (Fig 5), suggesting that a stable signaling complex is present on endosomes in mature neurons. The initiation of comets from these puncta indicates that endosomes are likely the key site where Wnt signaling proteins promote nucleation. Colocalization of tagged Golgi proteins with Rab5 suggests that the previous association between Golgi markers and nucleation could have been due to leakage into endosomes. In addition, the identification of plasma membrane proteins acting upstream of γTub in dendrites suggests a more general role for the Golgi in the cell body by controlling secretion of arr and fz.

Wnt signaling receptors have been classically studied at the plasma membrane, where they bind extracellular ligands that can be autocrine or paracrine in nature. A requirement for arr and fz upstream of γTub in dendrites suggests that a Wnt ligand is likely involved. Failure of neuronal wntless knockdown to reduce γTub-GFP at branch points (Fig 5B) favors the hypothesis that the ligand may be secreted from a neighboring cell. In the embryo, wingless (wg)/Wnt-1 is made in a patch of epithelial cells adjacent to developing dendritic arborization neurons and helps pattern dendrite orientation in ddaE [81]. It would be very interesting if surrounding cells influenced the microtubule cytoskeleton in mature neurons through fz and arr at the plasma membrane. This signaling pathway is particularly intriguing in the context of regeneration or during neurodegenerative disease. During axon regeneration, the initial injury response involves a nucleation-dependent increase in microtubule dynamics, which serves a neuroprotective role [29]. Modulating Wnt signaling could therefore influence neuroprotection in dendrites. In addition, we have found that this pathway is required during dendrite regeneration to position nucleation sites in regrowing dendrites [98]. Interestingly, G protein coupled receptors (GPCRs) represent 33% of all Food and Drug Administration–approved drug targets, and as part of this family, fz presents a possible target [82].

Local microtubule nucleation also occurs in axons [26, 83–85]. As Rab5 endosomes are present throughout axons, it will be interesting to determine whether Wnt signaling proteins can be recruited to axonal early endosomes and whether they recruit nucleation proteins in this part of the cell. It is also possible that a link between Wnt signaling, endosomes, and nucleation could exist more broadly in other cell types. Indeed, the localization of Axin to centrosomes [54, 55] suggests that even in mitotic cells, parts of this relationship are conserved. Intriguingly, endosomal membranes are concentrated around the centrosome [86], and Rab5 reduction disrupts mitosis [86–88], so it is possible that Wnt signaling proteins, endosomes, and nucleation function together at centrosomes.

## Methods

### *Drosophila* genetics and lines

Many stocks used in this study were from the Vienna Drosophila Resource Center or Bloomington Drosophila Stock Center (NIH P40OD018537). Refer to S1 Table for information on specific strains used as well as how they are referred to in figures. RNAi experiments were performed by crossing *Drosophila* strains with UAS-controlled hairpins to tester lines that included 221-Gal4 to drive expression in class I dendritic arborization neurons, fluorescently tagged markers, and UAS-Dcr2 to increase RNAi knockdown efficiency [89]. Of the two dorsal class I neurons, the ddaE cell was chosen for analysis because the shape of its dorsal comb-like dendrite makes it particularly sensitive to perturbation of microtubule polarity [31]. In whole-brain imaging experiments, expression was driven panneuronally with elav-Gal4. The mutant fz stock *fz*^*F31*^ was graciously sent by Dr. Paul Adler at the University of Virginia, and *fz*^*P21*^ was kindly sent by Dr. Yashi Ahmed at Dartmouth University. Constitutively active Gαs and inactive Gαo fly lines were given to us by Dr. Andrew Tomlinson at Columbia University Medical Center. Tester lines for screens include (UAS-Dcr2, mCD8-RFP; 221-Gal4, Apc2-GFP), (UAS-Dcr2, UAS-mCD8-RFP; 221-Gal4, UAS-Axin-GFP), (UAS-Dcr2, UAS-mCD8-RFP; 221-Gal4, UAS-dsh-GFP) (UAS-Dcr2, UAS-mCD8-RFP; 221-Gal4, UAS-γTub-GFP), (UAS-Dcr2, UAS-mCD8-RFP; 221-Gal4, UAS-Rab5-GFP) (UAS-Dcr2; 221-Gal4, UAS-EB1-GFP). Additional fly lines used were (221-Gal4, UAS-Mito-GFP), (221-Gal4, UAS-γTub-GFP), (221-Gal4, UAS-fz-EGFP), (221-Gal4, UAS-Axin-GFP) (IG1-Gal4, UAS-mCD8-RFP/cyo; FzR52/TM6), (UAS-gTub-GFP/cyo; FzP21/TM6), (UAS-Mito-RFP), (UAS-Axin-RFP-ActA), (UAS-dsh-GFP), (dsh-Clover) (mcherry-Rab5), (UAS-RAb4-mRFP), (Rab11-cherry), (UAS-ManII-EBFP), (UAS-ManII-EGFP), (UAS-GalT-YFP), (UAS-Rab5-GFP), (UAS-Rab5-YFP), and (elav-Gal4, EB1-RFP). Control genotypes were matched for UAS-driven transgene number with experimental genotypes. For all RNAi and overexpression experiments, the tester lines were crossed either to Rtnl2 (control 1) or γTub37C (control 2) RNAi lines (see S1 Table for stock numbers used). These two targets were chosen for controls because neither is expected to be expressed in neurons. Rtnl2 is thought to be a pseudogene, and γTub37C is the maternal γTub, as opposed to γTub23C, the somatic γTub referred to throughout the manuscript as γTub. For mutant experiments, tester lines we crossed to *yw* flies (control) did not contain any UAS-driven transgenes.

### Confocal in vivo microscopy

After mating virgin female flies from tester lines with RNAi male flies, embryos were collected on 35-mm caps filled with *Drosophila* cornmeal media every 24 hours. Caps with embryos/larvae were incubated for 3 days at 25 ^o^C, and live *Drosophila* third instar larvae were collected for mounting from these caps. After rinsing with water, individual larvae were mounted on a microscope slide with a circular piece of dried agar in the middle. Larvae were placed on the dried agar and whole mounted ventral side down by applying sublethal pressure with a coverslip (22 × 40 mm), which was then secured with tape. To locate larvae under the microscope, 10× objectives were used. To find ddaE neurons in segments a2-a4, 60× oil (NA 1.42) (Olympus) and 63× oil (NA 1.4) (Zeiss) objectives were used. For UAS-Apc2-GFP localization, larvae were imaged on an Olympus FluoView1000. For the rest of the fluorescent markers, including UAS-Mito-GFP, UAS-Mito-RFP, UAS-Axin-GFP, and UAS-γTub-GFP, larvae were imaged on an Olympus FluoView1000 or a Zeiss LSM800, as indicated in the figures. Markers including UAS-Rab4-mRFP, Rab11-cherry, mcherry-Rab5, UAS-ManII-EGFP, UAS-ManII-EBFP, UAS-GalT-YFP, and UAS-iBlueberry were imaged exclusively on the Zeiss LSM800. The UAS-dsh-GFP, UAS-Rab5-GFP, and experiments showing EB1 comets originating off Rab5 endosomes or wnt signaling protein puncta were imaged on the Zeiss LSM800. These markers include UAS-Rab5-YFP, UAS-Rab5-tdTomato, EYFP-Rab5, UAS-fz-EGFP, and dsh-Clover. The LSM800 is built on an AxioImager.Z2 and is operated with Zeiss Zen Blue software.

### Larval brain imaging

Transgenic elav-Gal4, EB1-RFP flies were crossed with flies expressing either UAS-Apc2-GFP or UAS-Axin-GFP. Embryos were grown at 25 ^o^C for 1 day, and first instar larvae were mounted using the same protocol as third instar. The brain was located using the 10× objective of an Olympus FluoView1000 microscope. One of the lobes was then examined using a 60× oil (NA 1.42) objective. Actively dividing neuroblasts were identified by the star-like pattern of EB1-RFP around spindle poles and their relatively large size compared with surrounding cells. Movies were acquired by collecting images every second for up to 350 seconds.

### Plasmid and *Drosophila* line construction

To generate Axin targeted to mitochondria, the region that encodes the short C-terminal ActA mitochondrial targeting sequence [66] was synthesized by Genscript and cloned via Clone EZ Technology downstream of tDimer-Red12 (RFP) [90] in pUAST to create a carboxyl-terminal fusion. The synthesized sequence was as follows: agatctagattaattcttgcaatgttagctattggcgtgttctctttaggggcgtttatcaaaattattcaattaagaaaaaataattaa.

The resulting vector, pUAST-RFP-ActA, was then linearized with SpeI and KpnI and gel isolated.

Herculase from Agilent (catalog #600675) and the following primers were used to PCR amplify the long isoform of Axin from *Drosophila* cDNA FI19317 (stock #1647293) obtained from the Drosophila Genomics Resource Center (https://dgrc.bio.indiana.edu/product/View?product=1647293):

Axin5′ primer: ctcgagggcgcgccaactagtATGAGTGGCCATCCATCGGGAATCCGGAAACATGATGATAATGAG

Axin3′ primer:

aggccggccacgcgtggtaccATCGGATGGCTTGACAAGACCCATCGCTTTGTC

The PCR product was gel isolated and cloned by In-Fusion (Clontech catalog #639646 In-Fusion HD Cloning System) into SpeI-KpnI linearized pUAST-RFP-ActA to create pUAST-Axin-RFP-ActA. To generate a pUAST-Axin-RFP vector lacking the ActA sequence, tDimer-Red12 (RFP) was subcloned from the original pUAST-RFP construct as an FseI-PsiI fragment, which was gel isolated and used to replace the FseI-PsiI fragment in pUAST-Axin-RFP-ActA by ligation.

To generate arr tagged with the mScarlet-I fluorescent protein (called for simplicity arr-RFP), Herculase from Agilent (catalog #600675) and the following primers were used to PCR amplify the entire *Drosophila* arr open reading frame from a pUAS-arrow construct generously provided by Marcel Wehrli [91]:

ArrFBglII

AATTGGGAATTCGTTAACAGATCTCAAAACATGGCTTTCGAGCCATACACAAAGTC G

ArrRAcc65I

AGCAGGCCGGCCACGCGTGGTACCCGTAAATCCCCGACTTGGCGACTGTACTGG

The PCR product was gel isolated and cloned by In-Fusion (Clontech catalog #639646. In-Fusion HD Cloning System) into gel-isolated pUAS-mScarlet-I [92] linearized by BglII-Acc65I producing C-terminally tagged arr-RFP. UAS-mScarlet-I was generated by inserting the mScarlet-I coding sequence into a pUAST backbone with an expanded polylinker.

pUAST-Rab5-tdTomato plasmid [93] was obtained from addgene and sent to BestGene for injection into *Drosophila* embryos. For simplicity, we call the flies UAS-Rab5-RFP. Transgene insertion sites were mapped to chromosomes using standard segregation techniques with balancer chromosomes.

pCasper4-Dsh::Clover2 was made by replacing EGFP of pCasper4-Dsh::EGFP [56] (Axelrod, 2001) with Clover2, a derivative of Clover [94]. In detail, the last 713 bp of Dsh coding sequence together with EGFP was cut out by XhoI/XbaI double digestion. Then, the same 713 bp of Dsh coding sequence without stop codon was cloned back into the XhoI/XbaI digested pCasper4-Dsh::EGFP to make a pCasper4-Dsh, introducing an XbaI site immediately following the Dsh coding sequence. Then, the 717 bp clover2 (with stop codon) fragment was cloned into the XbaI site in pCasper4-Dsh. The Clover2 fragment was PCR amplified from the vector pNCS-Clover2 (Michael Lin lab at Stanford).

UAS-dsh::GFP was generated by cloning the dsh cDNA from EcoRI in the 5′ UTR to EcoRI in the 3′ UTR into pUAST [95]. The GFP coding sequence was added by fusing the SnaBI site near the 3′ end of dsh to ClaI near the 5′ end of GFP by filled-in blunt ligation in pBS+beta. This fusion sequence was cloned into pCS2+, into which it was placed after the beta-globin 5′ UTR, and the GFP was substituted with EGFP. A fragment containing part of the beta-globin 5′ UTR, part of the dsh 5′ UTR, dsh cDNA::EGFP, followed by SV40 polyA, was cloned back into pUAST to generate the plasmid for injection. Plasmid injections into *Drosophila* embryos were performed by BestGene, and transgene insertion sites were mapped to chromosomes using standard segregation techniques with balancer chromosomes.

pUAST-γTub-TagRFPT was generated by first digesting GFP from the pUAST-γTub-GFP plasmid created in our previous publication [20] and digesting TagRFPT from pUAST-EB1-RFPT, also previously generated in our lab (Feng and colleagues [77]), using EcoRI and KpnI sites. TagRFPT was then ligated into the pUAST-γTub backbone. The product was then confirmed with diagnostic digest and sent to BestGene for plasmid injections into *Drosophila* embryos. Transgene insertion sites were mapped to chromosomes using standard segregation techniques with balancer chromosomes.

### Microtubule polarity assay

Movies of EB1-GFP in the dorsal comb dendrite of the ddaE neuron were acquired with an AxioCam M2 or AxioCam 506 on a Zeiss ImagerM2 microscope running Zen Blue in live *Drosophila* third instar larvae. A Colibri2 LED illumination system was used to excite GFP with 470-nm light, and a 63× 1.4-NA objective was used. Movies were acquired for 300 frames at a rate of one frame per second. After acquisition, the Template Matching and Slice Alignment plug-ins in Fiji were used for stabilization. EB1-GFP comets visible for at least three frames were classified as growing toward or away from the cell body. The main trunk of the comb dendrite, distal to the first branch point, was used for analysis. Kymographs were generated using a built-in Fiji plug-in. Data from each cell of a given genotype were pooled to generate total numbers of comets moving toward or away from the cell body. Statistical analysis was conducted using logistic regression.

### Spawning assay and quantification

EB1-GFP movies were acquired at one frame per second for 300 seconds. Most EB1-GFP movies were acquired with a Zeiss widefield microscope. When EB1-GFP was paired with mito-RFP or Axin-RFP-ActA, movies were acquired on a Zeiss confocal LSM800 microscope. For all movies, the main trunk of the comb dendrite was used for analysis. Spawning events were characterized as emergence of an EB1 comet that covers a distance of at least 1 μm. Events that began before the movie started were not counted as a spawn event. Comet events that started off view and polymerized into the region of interest were also not counted. The same parameters were used for comets originating form labeled endosomes or Wnt proteins. Length measurements were performed in Fiji with the segmented line tool for non–branch point areas along the main trunk. For branch points, length was calculated by drawing a line from the start of the taper on the side of the branch furthest from the cell body to the end of the taper closest to the cell body. Total comet counts were then divided by total length of branch point or non–branch point alike to produce a normalized value of comet number per micrometer per 300 seconds.

### Axon injury microtubule dynamics assay

A Micro-Point pulsed UV laser (Andor Technology) focused through the 63× objective of a Zeiss LSM800 microscope was used to make a precise cut to the proximal axon of a ddaE neuron in a third instar larvae. Larvae were then incubated at 20 ^o^C for 8 hours, and the comb dendrite of the injured cell was imaged using a AxioCam M2 or AxioCam 506 on a Zeiss ImagerM2. EB1-GFP movies were acquired in the same way as for the microtubule polarity assay. Images were stabilized using the Template Matching and Slice Alignment plug-in in Fiji. Total comet number was then counted in Fiji and normalized to the length of the dendrite region analyzed. This produced a value of microtubule comets per micrometer. Within Fig 4, data shown in (B) were generated slightly differently than that in (E) and (F). The person who produced (B) used a defined 10 μm of length in the dendrite as opposed to a variable length of 20–30 μm in (E) and (F). The set 10-μm short distance has the probability of many more comets passing through during the same time interval as EB1 comets have a long run length. This explains why the two data sets are slightly different in value. Kymographs were generated using a built-in Fiji plug-in. Statistics were generated using a linear regression model.

### EB1 comet initiation off endosome assays

Videos were acquired at one frame per second for 300 seconds on a Zeiss LSM800 microscope. Only branch points in focus during the duration of the video were used for quantification. Comets that originated de novo at branch points off of either Rab5 or Wnt proteins were counted. Comets were not counted if they originated from a growing minus end. They also were not counted if the comet was a result of a discernable catastrophe/rescue event. For the experiment involving dsh-decorated Rab5 endosomes, the same stipulations were followed. Total number of Rab5 puncta were counted and divided into two categories. These consisted of dsh-positive and dsh-negative Rab5 endosomes. The percentage of dsh-decorated Rab5 endosomes was calculated using Fiji, and comet events were also visualized with the software.

### Fillet prep and immunostaining

Third instar larvae expressing UAS-mcd8-RFP;221 Gal4 to label class I da neurons were dorsally filleted in Schneider’s media using dissection scissors after pinning both head and tail down with 0.10-mm steel insect pins. After making a longitudinal incision between the primary trachea, the gut and trachea were carefully removed, leaving only muscle and skin. Following this removal, four additional pins were used to carefully stretch and pin the larval body wall down. Immediately following this, the medium was removed, and 4% PFA was used to fix the larvae for 30 minutes. After fixation, cells in the larvae were permeabilized in 3% PBS TX100 for 15 minutes. After this, the fillets were moved to blocking solution of 10% NGS, 2% BSA, and 0.2% TX100 in PBS for 1 hour. Following this, larvae were incubated overnight at 4°C in a 1:100 solution of an antibody targeting Axin generated and described in Wang and colleagues [42]. The following day, the Axin antibody was washed off 5 times for 5 minutes each. The fillets were then exposed to a secondary 488 goat anti-guinea antibody at 1:500 for 2 hours. Lastly, the secondary antibody was washed off 5 times for 5 minutes each, and then larvae were imaged using a Zeiss LSM800 microscope using the same mounting procedure as the live larvae. The same procedure was followed for staining with the acetylated tubulin antibody obtained from Sigma (T7451). The only difference was that 1:500 was used for primary antibody, and 1:1000 was used for secondary antibody.

### Fluorescence intensity quantification methods

For branch point intensity measurements, z-stack images were acquired with either an Olympus or Zeiss confocal microscope as noted on each graph, and images were prepared and quantified using the image processing software Fiji (ImageJ). Maximum-projection stacked images were used for all localization analyses. UAS-Apc2-GFP was measured in a binary manner by scoring each branch point as Apc2-GFP present or absent. Similarly, for UAS-dsh-GFP, any branch point that had a distinct punctum of GFP was counted as present. For UAS-γTub-GFP and UAS-Axin-GFP, the regions between branch points (non–branch points) and within branch points along the dorsal comb dendrite were manually outlined, and average pixel intensities were measured (S1 Fig). Typically, 8–12 branch points and non–branch point regions were outlined in each cell, and a single average branch point and non–branch point value was generated for the cell. These two values were then subtracted from each other to determine how much more fluorescence accumulated at the branch point than in between. Refer to S1 Fig for an example image with manual branch point and non–branch point outlines. To normalize raw fluorescence intensities, the average branch point–non–branch point value was divided by 100 to generate a normalization constant, which was then multiplied to each raw fluorescence intensity value, including those of the control. This generated an average for the control close to but not exactly 100 because of rounding errors in Excel. For information about cytoplasmic GFP quantitation, see S1G Fig. Axin-GFP values were normalized in the same way.

For mitochondrial colocalization experiments, an overview z-stack image using sequential channel scanning was collected on a Zeiss LSM800 Imager.Z2 running Zen Blue, and maximum-intensity projections were generated using Fiji. Secondary dendrites emerging from the trunk of the dorsal comb dendrite of the ddaE neuron were chosen as regions for analysis because mitochondria are found along their length, but little Axin normally concentrates there. A segmented line was generated along one of these dendrites, and fluorescent intensity line tracings were made using Fiji for both red and green channels. To normalize these intensities, the values along the line were divided by the highest pixel value, and these values were plotted. For the correlation analysis, the Fiji plug-in JACoP was used to calculate a Pearson’s coefficient (R score) [96]. This coefficient was calculated for the entire comb dendrite between red and green channels for each of the four conditions.

### Class I dendrite morphology analysis

Images were acquired on a Zeiss LSM700 confocal microscope using a 63× oil objective NA 1.2 running Zen Blue. Images were processed in Fiji as maximum-intensity projections. To quantify morphology, branch points of all dendritic processes were counted and summed as previously described [97]. Images used for quantification were collected one cell per animal.

### Statistical methods

Multiple linear regression analysis was used to compare conditions against a control for all localization experiments and for microtubule dynamics. This analysis calculates *p*-values the same way as ANOVA, but it allowed us to specify which condition was the control. A logistic regression was used to compare polarity assay conditions to a control because this is a pooled data set. GraphPad Prism 6 software was used to carry out statistical analyses. See individual figure legends for the statistical test used. Statistical significance is noted as *p < 0.05, **p < 0.01, and ***p < 0.001. Statistical methods were carried out after consulting with Haley Brittingham, a master’s student in the Penn State Statistics Department. In all figures with error bars, they represent standard deviation.

## Supporting information

S1 Fig Candidate screen for proteins required for γTub-GFP localization to dendrite BPs. (A) Example images of γTub-GFP and mCD8-RFP in ddaE neurons are shown with BP (left) and nBP (right) regions outlined. Outlines were drawn manually in Fiji, and the measuring tool was then used to measure fluorescence in each region. These values were then averaged for each cell. The BP was outlined until it began to taper, and this was the cutoff point for where the nBP areas began. (B–E) Quantification of γTub-GFP at BPs is shown in larvae expressing different RNAi hairpins. Control 1 is Rtnl2 RNAi, as Rtnl2 is thought to be a pseudogene. Control 2 is γTub37C RNAi. This isoform of γTub is maternally deposited and not expressed in somatic cells like neurons. Values were generated by subtracting mean nBP fluorescence from BP fluorescence for each cell; normalized fluorescence values are shown. Shaded colors over x-axis names indicate which functional groups the RNAi lines belong to and are noted as pink for mitochondria, yellow for Ankyrin2 and Neuroglian, and purple for branched-actin regulators. (F) Representative images showing a soluble cytoplasmic GFP (left) and UAS-γTub-GFP (right) under 221-GAL4. (G) Raw quantification of fluorescence showing BP and nBP values of gTub-GFP. The raw peak values (BP) for cytoplasmic GFP (BL 6658) were slightly dimmer than γTub-GFP, so they were multiplied by 1.1 to make the values easier to compare. Black bars represent either BP or nBP values for each condition. Gray bars indicate the subtraction of nBP from BP. A normalization constant was generated by setting the raw fluorescence value of BP-nBP to 100 for γTub-GFP. This constant was then used to normalize each γTub-GFP sample from every other genotype (Figs 1, 5 and S7). The number of cells (one per animal) is shown on the bars. Refer to S1 Table for all genotypes and S1 Data for data used to generate graphs in (B–E) and (G). γTub, γTubulin; dda, BP, branch point; dorsal dendritic arborization; GFP, green fluorescent protein; nBP, non–branch point; RFP, red fluorescent protein; RNAi, RNA interference; Rtnl2, reticulon 2; UAS, upstream activating sequence.
(TIF)

S2 Fig Endogenous arm, gish, Axin, and γTub localization in neurons. (A and B) Example images from a region of a third instar larval body wall are shown from animals expressing UAS-mCD8-RFP under the control of 221-GAL4 and either arm-GFP or gish-GFP under the control of their native promoters. (C) An example image from a filleted larva, immunostained with an antibody against Axin. Cell shape marker is the cytoplasmic marker UAS-iBlueberry, pseudocolored in red for viewing convenience. (D) Example image from the main trunk of a comb dendrite from an animal expressing UAS-mCD8-RFP under the control of 221-GAL4 and a CRISPR-tagged γTub-sfGFP at the endogenous locus. An orange arrow points to the branch point shown in the insets. γTub, γTubulin; arm, armadillo; GFP, green fluorescent protein; gish, gilgamesh; RFP, red fluorescent protein; sfGFP, super-folder green fluorescent protein; UAS, upstream activating sequence.
(TIF)

S3 Fig Class I dendrite morphology is unaltered when gTub or Wnt signaling proteins are reduced. (A) Example images of class I da neurons expressing either control or gTub23C RNAi hairpins. The number of total dendrite branch points for each example is provided. (B) Quantification of total branch point number for RNAi conditions. Sample size is shown in the bars and represents number of cells quantified for each condition. Branch point number was summed between all dendritic processes, and a linear regression was used to determine statistical significance. *p < 0.05, **p < 0.01, ***p < 0.001. Refer to S1 Table for all genotypes and S1 Data for data used to generate the graph in (B). da neuron, dendritic arborization neuron; RNAi, RNA interference.
(TIF)

S4 Fig Microtubule density is not reduced with Wnt signaling protein knockdown. (A) Example images from filleted larva, immunostained with an antibody against acetylated tubulin (right panels). Cell shape marker is the membrane marker UAS-mCD8-GFP (left panels). The top example is control, and the bottom is Axin A RNAi. A white arrow in the red panel indicates a 10-μm region that was used for quantification of acetylated tubulin fluorescence. The inset in each panel is an enlarged view of this region. (B) Quantification of fluorescence intensity of acetylated tubulin in the first proximal 10 μm of the comb dendrite. Sample size is shown in the bars and represents the number of cells quantified. A linear regression was used to determine statistical significance. *p < 0.05, **p < 0.01, ***p < 0.001. Refer to S1 Table for all genotypes and S1 Data for data used to generate the graph in (B). GFP, green fluorescent protein; RNAi, RNA interference; UAS, upstream activating sequence.
(TIF)

S5 Fig RNAi’s targeting γTub, cnn, and Plp do not affect Apc2-GFP localization to branch points. (A) Example image of Apc2-GFP and mCD8-RFP in ddaE neurons expressing UAS-Rtnl2 RNAi (control 1) (VDRC 33320) and UAS-γTub23C RNAi (VDRC 19130) hairpins are shown. Orange arrows indicate branch points with high Apc2-GFP signal, scored as occupied. Insets in the top corner of each image indicate the top branch point, indicated by an arrow. (B) Quantification of Apc2-GFP branch point occupancy is shown in neurons expressing different RNAi hairpins. Refer to S1 Table for all genotypes and S1 Data for data used to generate the graph in (B). γTub, γTubulin; Apc, adenomatous polyposis coli; cnn, centrosomin; dda, dorsal dendritic arborization; GFP, green fluorescent protein; Plp, Pericentrin-like protein; RFP, red fluorescent protein; RNAi, RNA interference; Rtnl2, reticulon 2; UAS, upstream activating sequence; VDRC, Vienna Drosophila Resource Center.
(TIF)

S6 Fig dsh localizes to endogenous Rab5 early endosomes. (A) Example image of endogenous mcherry-Rab5 and UAS-dsh-GFP. Because there is no membrane marker, the panel on the right was enhanced and used as a template to draw the outline of the dendrite branch point. (B) Two example images of a section of dendrite where the colocalization between mcherry-Rab5 and dsh-Clover can be seen in the branch point of the neuron. The orange arrow indicates the colocalization. The middle and right panels show the green and red channels, with orange arrows indicating colocalization. dsh, dishevelled; GFP, green fluorescent protein; UAS, upstream activating sequence.
(TIF)

S7 Fig The Golgi is not required for γTub-GFP localization, and other Rabs do not colocalize with dsh-GFP. (A) Three examples of dendritic localization of Golgi markers, including UAS-ManII-eBFP, UAS-ManII-eGFP, and UAS-GalT-YFP, and one example of the early endosomal marker UAS-Rab5-YFP. The UAS-ManII-eBFP (pseudocolored) is coexpressed with UAS-Rab5-GFP. All other markers have the cell shape marker UAS-mCD8-RFP coexpressed for localization reference. (B) Quantification of γTub-GFP at BPs is shown in larvae expressing different RNAi hairpins targeting Golgi-associated proteins. Values were generated by subtracting mean nBP fluorescence from BP fluorescence for each cell; normalized fluorescence values are shown. The number of cells (one per animal) is shown on the bars. Error bars indicate standard deviation. (C) Quantification of EB1-GFP comet direction in the main trunk of the ddaE dendrite in animals expressing hairpin RNAi’s. The percentage of microtubules oriented plus-end-out is plotted as a summed value across all cells for each genotype. The numbers on each bar are total EB1-GFP comets counted, and at least 15 cells were analyzed for each genotype, with one cell per animal. A logistic regression was used to determine significance. *p < 0.05, **p < 0.01, ***p < 0.001. (D) Example image of endogenous Rab11-cherry and UAS-dsh-GFP. Because there is no membrane marker, the panel on the right was enhanced and used as a template to draw the outline of the dendrite branches. (E) Examples images of either EYFP-Rab5 or UAS-Rab5-GFP coexpressed with UAS-ManII-eBFP. The orange arrows point to puncta of colocalization between the two markers in each case. Insets in the top corner of each image show the example highlighted with an arrow in each image. For EYFP-Rab5, the bottom arrow correlates with the bottom left insets. Refer to S1 Table for all genotypes and S1 Data for data used to generate graphs in (B) and (C). γTub, γTubulin; BP, branch point; dda, dorsal dendritic arborization; dsh, dishevelled; EB1, end-binding protein 1; eGFP, enhanced green fluorescent protein; EYFP, enhanced yellow fluorescent protein; GFP, green fluorescent protein; MannII, mannosidase-II; nBP, non–branch point; RFP, red fluorescent protein; RNAi, RNA interference; UAS, upstream activating sequence.
(TIF)

S8 Fig Axin colocalizes with a subset of Rab5 endosomes in the cell body and can recruit a diffuse gTub to puncta at branch points. (A) Images showing the localization of UAS-Rab5-RFP in the cell body of a ddaE neuron when coexpressed with UAS-Axin-GFP. Orange arrows indicate the subset of Rab5 endosomes that colocalize with Axin-GFP. (B) Images show the localization of UAS-γTub-RFP (middle) in the branch points of a ddaE neuron when coexpressed with UAS-Axin-GFP (left) using the 221-Gal4 driver. Merged channel is provided on the right. Orange arrow points to a colocalized puncta at the branch point and is enlarged in the inset. γTub, γTubulin; dda, dorsal dendritic arborization; GFP, green fluorescent protein; RFP, red fluorescent protein; UAS, upstream activating sequence.
(TIF)

S9 Fig Microtubules initiate from dsh puncta. (A) Example five-frame stills of microtubule comet formation off UAS-dsh-GFP puncta. The top example is shown coexpressed with UAS-EB1-GFP, and the bottom is with UAS-EB1-TagRFPT. In both examples, the first frame includes a blue arrow to show the dsh-GFP puncta that the microtubule comet will initiate off. Subsequent frames track movement of the microtubule with a white arrow. Time stamp at the top-right corner correlates to the time point in the corresponding S8 and S12 Movies. dsh, dishevelled; EB1, end-binding protein 1; GFP, green fluorescent protein; UAS, upstream activating sequence.
(TIF)

S10 Fig Axin is sufficient to recruit cnn to ectopic cellular sites. (A and B) UAS-cnn-GFP was coexpressed with either UAS-mito-RFP or UAS-Axin-RFP-ActA using 221-Gal4. Overview images of the entire dendrite arbor are shown. (C and D) Regions within the comb dendrite indicated by the dashed lines in (A) and (B). (E and F) Fluorescence intensity measurements from the regions shown in (C) and (D). (G) A plot of Pearson’s correlation coefficient between the two conditions. The y-axis indicates the R score, with 1 being positive correlation, 0 meaning no correlation, and −1 meaning negative correlation. The key to the right of the graph indicates which conditions match the symbols. (H) A diagram of the chimeric protein used to tag Axin with RFP (tdimer2[12]) and target it to mitochondria is shown. Refer to S1 Table for all genotypes and S1 Data for data used to generate graphs in (E), (F), and (G). ActA, actin assembly promoting protein A; cnn, centrosomin; GFP, green fluorescent protein; Mito, mitochondrial; RFP, red fluorescent protein; UAS, upstream activating sequence.
(TIF)

S11 Fig Analysis of microtubule spawning events in dendrites with reduced and ectopic Axin. (A) Example images compiled from EB1-GFP movies of the main comb dendrite trunk were generated using a summed projection of all 300 frames. Movies were acquired in control (*yw*) and *Axin*^*18*^*/+* mutant backgrounds. White arrows indicate spawning events at branch points, and red arrows indicate comets that spawn from non–branch point regions during the 300-second movie. (B) Quantification of number of EB1 comets per micrometer for the entire 300-second movie is shown. (C) UAS-EB1-GFP was coexpressed with either RFP-tagged mitochondria or the chimeric UAS-Axin-RFP-ActA. Summed example images are shown. Arrows follow the same scheme as the top two panels. (D) Quantification of number of EB1 comets per micrometer for the entire 300-second movie is shown. (E) Kymographs generated from neurons expressing either dsh-clover and UAS-EB1-GFP or UAS-Axn-RFP and UAS-EB1-GFP. dsh-clover is used as the control because it is endogenously tagged and thus is not overexpressed like the Axin-RFP. (F and G) Microtubule polarity and dynamics of control versus UAS-Axin-RFP. Sample size shown in or above the bars represents the number of cells imaged; except in the case of polarity, it is the number comets. Error bars indicate standard deviation. A linear regression was used for dynamics, and a logistic regression was used for polarity to determine statistical significance. *p < 0.05, **p < 0.01, ***p < 0.001. Refer to S1 Table for all genotypes and S1 Data for data used to generate graphs in (B), (D), (F), and (G). ActA, actin assembly promoting protein A; Axn, Axin; dsh, dishevelled; EB1, end-binding protein 1; GFP, green fluorescent protein; RFP, red fluorescent protein; UAS, upstream activating sequence; *yw*, *yellow*, *white*.
(TIF)

S1 Data Quantitation for all graphs is included in the Excel file. The relationship between individual tabs and figures is noted in the title of the tab. Additional information about which data relate to a particular panel in a figure is included in the data sheet.
(XLSX)

S1 Table *Drosophila* stocks and other reagents used in the study are documented in this table. (XLSX)

S1 Movie Axin localizes to the centrosome of dividing neuroblasts in whole larval brains. UAS-Axin-GFP and UAS-EB1-RFP were coexpressed in neuroblasts using the pan neuronal driver elav-Gal4. First instar larvae were imaged as described in the methods. EB1, end-binding protein 1; GFP, green fluorescent protein; RFP, red fluorescent protein; UAS, upstream activating sequence.
(MOV)

S2 Movie Microtubule polarity is disrupted when proteins required for γTub localization to branch points are reduced. Movies of EB1-GFP in the dorsal comb dendrite were acquired by taking an image every second for 300 seconds. Examples of two movies are shown side by side. The cell body is oriented at the bottom of the image for both movies. A neuron expressing Rtnl2 RNAi (control 1) (VDRC 33320) is shown on the left, and a neuron expressing Axin RNAi (VDRC 7748) is on the right. Kymographs in Fig 6 were generated from these movies. γTub, γTubulin; EB1, end-binding protein 1; GFP, green fluorescent protein; RNAi, RNA interference; Rtnl2, reticulon 2; VDRC, Vienna Drosophila Resource Center.
(MOV)

S3 Movie The increase in microtubule dynamics following axotomy is blocked when proteins required for γTub localization are reduced. Axons of ddaE neurons were severed with a pulsed UV laser, and EB1-GFP movies were acquired from the comb dendrite 8 hours later. The cell body is oriented at the bottom of the image for both videos. The cell on the left expresses Rtnl2 RNAi (control 1) (VDRC 33320), and the one on the right expresses Axin RNAi (VDRC 7748) hairpins. Kymographs in Fig 7 were generated from these movies. γTub, γTubulin; dda, dorsal dendritic arborization; EB1, end-binding protein 1; GFP, green fluorescent protein; RNAi, RNA interference; Rtnl2, reticulon 2; VDRC, Vienna Drosophila Resource Center.
(MOV)

S4 Movie EB1 comets initiate from Rab5 endosomes at dendrite branch points. The three examples include UAS-EB1-GFP with UAS-Rab5-YFP, UAS-EB1-TagRFPT with EYFP-Rab5, and UAS-EB1-GFP with UAS-Rab5-tdTomato. During the movies, red arrows mark the endosome, and white arrows mark and follow microtubule plus-end growth. EB1, end-binding protein 1; EYFP, enhanced yellow fluorescent protein; GFP, green fluorescent protein; RFP, red fluorescent protein; UAS, upstream activating sequence.
(MOV)

S5 Movie EB1 comets initiate from Rab5 endosomes at dendrite branch points. The three examples include UAS-EB1-GFP with UAS-Rab5-YFP, UAS-EB1-TagRFPT with EYFP-Rab5, and UAS-EB1-GFP with UAS-Rab5-tdTomato. During the movies, red arrows mark the endosome, and white arrows mark and follow microtubule plus-end growth. EB1, end-binding protein 1; EYFP, enhanced yellow fluorescent protein; GFP, green fluorescent protein; RFP, red fluorescent protein; UAS, upstream activating sequence.
(MOV)

S6 Movie EB1 comets initiate from Rab5 endosomes at dendrite branch points. The three examples include UAS-EB1-GFP with UAS-Rab5-YFP, UAS-EB1-TagRFPT with EYFP-Rab5, and UAS-EB1-GFP with UAS-Rab5-tdTomato. During the movies, red arrows mark the endosome, and white arrows mark and follow microtubule plus-end growth. EB1, end-binding protein 1; EYFP, enhanced yellow fluorescent protein; GFP, green fluorescent protein; RFP, red fluorescent protein; UAS, upstream activating sequence.
(MOV)

S7 Movie EB1 comets initiate from fz puncta. The example includes UAS-EB1-GFP and UAS-fz-eGFP. During the movie, red arrows mark the fz punctum, and white arrows mark and follow microtubule plus-end growth. EB1, end-binding protein 1; eGFP, enhanced green fluorescent protein; fz, frizzled; GFP, green fluorescent protein; UAS, upstream activating sequence.
(MOV)

S8 Movie EB1 comets initiate from dsh puncta. The two examples include UAS-EB1-GFP with UAS-dsh-GFP and UAS-EB1-GFP with dsh-clover. During the movies, red arrows mark the dsh punctum, and white arrows mark and follow microtubule plus-end growth. dsh, dishevelled; EB1, end-binding protein 1; GFP, green fluorescent protein; UAS, upstream activating sequence.
(MOV)

S9 Movie EB1 comets initiate from dsh puncta. The two examples include UAS-EB1-GFP with UAS-dsh-GFP and UAS-EB1-GFP with dsh-clover. During the movies, red arrows mark the dsh punctum, and white arrows mark and follow microtubule plus-end growth. dsh, dishevelled; EB1, end-binding protein 1; GFP, green fluorescent protein; UAS, upstream activating sequence.
(MOV)

S10 Movie EB1 comets initiate from Axin puncta. The example includes UAS-EB1-GFP and UAS-Axin-RFP. During the movie, red arrows mark the Axin punctum in the beginning, and white arrows mark and follow microtubule plus-end growth. EB1, end-binding protein 1; GFP, green fluorescent protein; RFP, red fluorescent protein; UAS, upstream activating sequence.
(MOV)

S11 Movie EB1 comets initiate from arr puncta. The example includes UAS-EB1-GFP and UAS-arr-mScarlet. During the movie, red arrows mark the arr punctum in the beginning, and white arrows mark and follow microtubule plus-end growth. arr, arrow; EB1, end-binding protein 1; GFP, green fluorescent protein; UAS, upstream activating sequence.
(MOV)

S12 Movie EB1 comets initiate from dsh puncta 2. The example includes UAS-EB1-TagRFPT and UAS-dsh-GFP. During the movie, red arrows mark the dsh punctum in the beginning, and white arrows mark and follow microtubule plus-end growth. The settings used for these videos were optimized so that EB1-TagRFPT comets could be visualized. The construct is very dim and needs both simultaneous scanning with a higher laser intensity. Therefore, there is bleed through of the red and green signal at the dsh puncta. This does not change the interpretation of the comet that comes off the dsh puncta because this is not colabeled. For colocalization experiments, sequential scans were taken to ensure no bleed through of the markers used. dsh, dishevelled; EB1, end-binding protein 1; GFP, green fluorescent protein; UAS, upstream activating sequence.
(MOV)

S13 Movie EB1 comets preferentially initiate from dsh-decorated Rab5 endosomes. The example movies include animals expressing UAS-EB1-GFP, UAS-dsh-GFP, and UAS-Rab5-RFP. During the movies, red arrows mark the dsh-GFP/Rab5-RFP puncta, and white arrows mark and follow the microtubule plus-end growth. dsh, dishevelled; EB1, end-binding protein 1; GFP, green fluorescent protein; RFP, red fluorescent protein; UAS, upstream activating sequence.
(MOV)

S14 Movie EB1 comets preferentially initiate from dsh-decorated Rab5 endosomes. The example movies include animals expressing UAS-EB1-GFP, UAS-dsh-GFP, and UAS-Rab5-RFP. During the movies red arrows mark the dsh-GFP/Rab5-RFP puncta, and white arrows mark and follow the microtubule plus-end growth. dsh, dishevelled; EB1, end-binding protein 1; GFP, green fluorescent protein; RFP, red fluorescent protein; UAS, upstream activating sequence.
(MOV)

S15 Movie Localization of γTub to mitochondria results in multiple EB1 initiation events from mitochondria. Movies of EB1-GFP were taken while coexpressing Axin-RFP-ActA. In rare events such as the example, multiple microtubules can be seen coming off mitochondria at the same time. Arrows indicate where the spawn point occurs and follows the three EB1-GFP comets as they travel off in an “aster-like” pattern. γTub, γTubulin; ActA, actin assembly promoting protein A; EB1, end-binding protein 1; GFP, green fluorescent protein; RFP, red fluorescent protein.
(MOV)

## Abbreviations

+TIP: microtubule plus end tracking protein
γTub: γTubulin
γTuRC: γTub ring complex
γTuSC: γTub small complex
ActA: actin assembly promoting protein A
Ank2: Ankyrin 2
Apc: adenomatous polyposis coli
arm: armadillo
Arpc4: actin related protein c4
arr: arrow
ATPsynβ: ATP Synthase β
Axn: Axin
BDSC: Bloomington Drosophila Stock Center
CAMSAP: calmodulin-regulated spectrin-associated protein
chb: chromosome bows
CLASP: cytoplasmic linker associated protein
CK1: casein kinase I
CLIP-190: cytoplasmic linker protein-190 kDa
cnn: centrosomin
Cyto: cytoplasmic
da neuron: dendritic arborization neuron
dda: dorsal dendritic arborization
Df: deficiency
dsh: dishevelled
EB1: end-binding protein 1
EGFP: enhanced green fluorescent protein
EYFP: enhanced yellow fluorescent protein
fz: frizzled
Gα: G protein alpha subunit
GDP: guanosine diphosphate
GFP: green fluorescent protein
gish: gilgamesh
GPCR: G protein coupled receptor
GSK3β: glycogen synthase kinase 3 beta
Grip: gamma tubulin ring protein, GTP, guanosine triphosphate
LRP: low-density lipoprotein receptor-related protein
lva: lava lamp
ManII: mannosidase-II
Miro: mitochondrial rho
Mito: mitochondrial
MTOC: microtubule organizing center
Nrg: Neuroglian
PCP: planar cell polarity
Plp: Pericentrin-like protein
rept: reptin
RFP: red fluorescent protein
RNAi: RNA interference
Rtnl2: reticulon 2
sfGFP: super-folder green fluorescent protein
sgg: shaggy
stan: starry night
UAS: upstream activating sequence
Vang: Van Gogh
VDRC: Vienna Drosophila Resource Center
wg: wingless
yw: *yellow*, *white*
