# Supplementary figures and images for: Endosomal Wnt signaling proteins control microtubule nucleation in dendrites
Source: PLoS Biol. 2020 Mar 12;18(3):e3000647. doi: 10.1371/journal.pbio.3000647 (PMC7067398; doi:10.1371/journal.pbio.3000647)

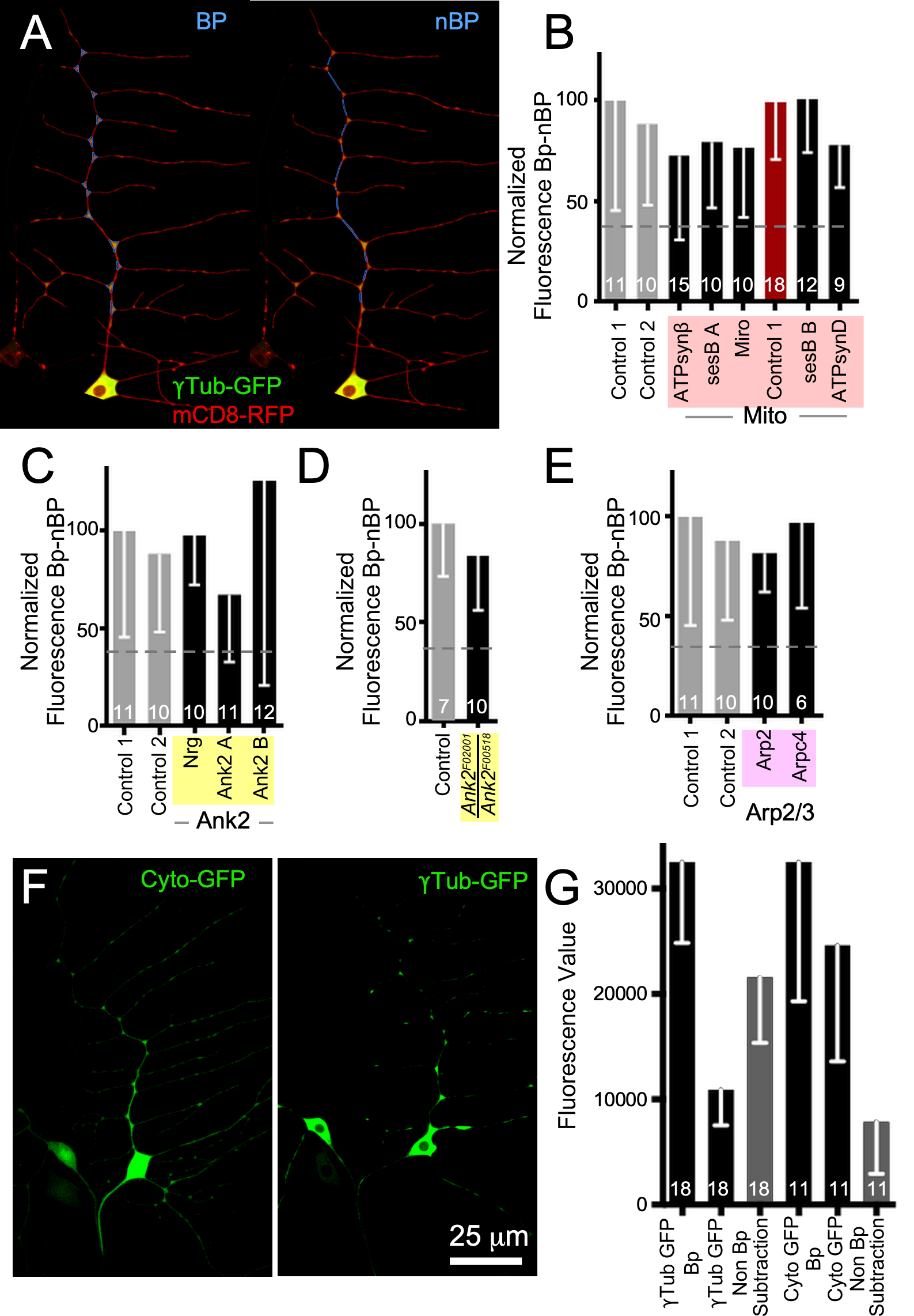

Supplement: S1 Fig — (A) Example images of γTub-GFP and mCD8-RFP in ddaE neurons are shown with BP (left) and nBP (right) regions outlined. Outlines were drawn manually in Fiji, and the measuring tool was then used to measure fluorescence in each region. These values were then averaged for each cell. The BP was outlined until it began to taper, and this was the cutoff point for where the nBP areas began. (B–E) Quantification of γTub-GFP at BPs is shown in larvae expressing different RNAi hairpins. Control 1 is Rtnl2 RNAi, as Rtnl2 is thought to be a pseudogene. Control 2 is γTub37C RNAi. This isoform of γTub is maternally deposited and not expressed in somatic cells like neurons. Values were generated by subtracting mean nBP fluorescence from BP fluorescence for each cell; normalized fluorescence values are shown. Shaded colors over x-axis names indicate which functional groups the RNAi lines belong to and are noted as pink for mitochondria, yellow for Ankyrin2 and Neuroglian, and purple for branched-actin regulators. (F) Representative images showing a soluble cytoplasmic GFP (left) and UAS-γTub-GFP (right) under 221-GAL4. (G) Raw quantification of fluorescence showing BP and nBP values of gTub-GFP. The raw peak values (BP) for cytoplasmic GFP (BL 6658) were slightly dimmer than γTub-GFP, so they were multiplied by 1.1 to make the values easier to compare. Black bars represent either BP or nBP values for each condition. Gray bars indicate the subtraction of nBP from BP. A normalization constant was generated by setting the raw fluorescence value of BP-nBP to 100 for γTub-GFP. This constant was then used to normalize each γTub-GFP sample from every other genotype (Figs 1, 5 and S7). The number of cells (one per animal) is shown on the bars. Refer to S1 Table for all genotypes and S1 Data for data used to generate graphs in (B–E) and (G). γTub, γTubulin; dda, BP, branch point; dorsal dendritic arborization; GFP, green fluorescent protein; nBP, non–branch point; RFP, red fluorescent prote [file pbio.3000647.s001.tif]

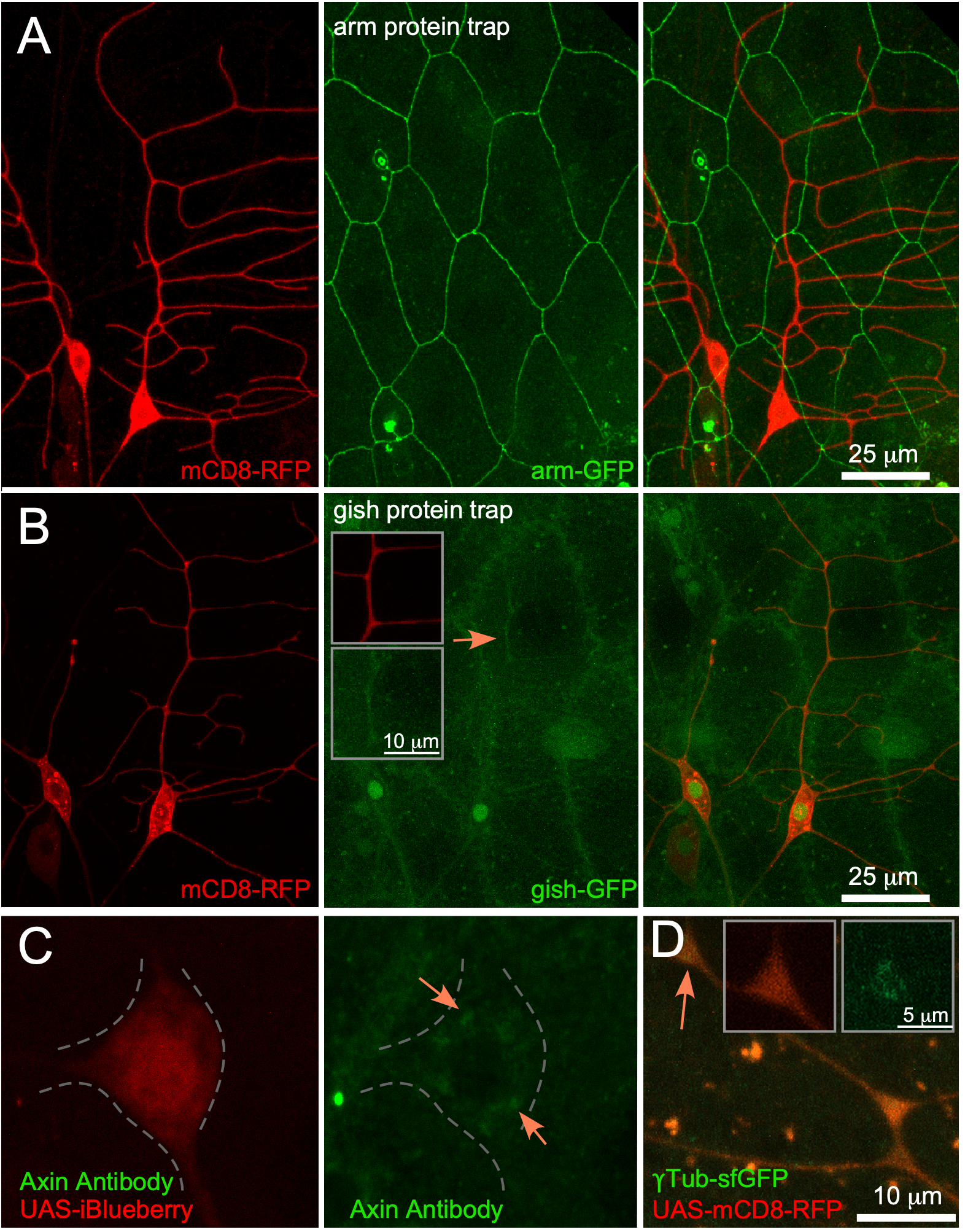

Supplement: S2 Fig — (A and B) Example images from a region of a third instar larval body wall are shown from animals expressing UAS-mCD8-RFP under the control of 221-GAL4 and either arm-GFP or gish-GFP under the control of their native promoters. (C) An example image from a filleted larva, immunostained with an antibody against Axin. Cell shape marker is the cytoplasmic marker UAS-iBlueberry, pseudocolored in red for viewing convenience. (D) Example image from the main trunk of a comb dendrite from an animal expressing UAS-mCD8-RFP under the control of 221-GAL4 and a CRISPR-tagged γTub-sfGFP at the endogenous locus. An orange arrow points to the branch point shown in the insets. γTub, γTubulin; arm, armadillo; GFP, green fluorescent protein; gish, gilgamesh; RFP, red fluorescent protein; sfGFP, super-folder green fluorescent protein; UAS, upstream activating sequence. (TIF) [file pbio.3000647.s002.tif]

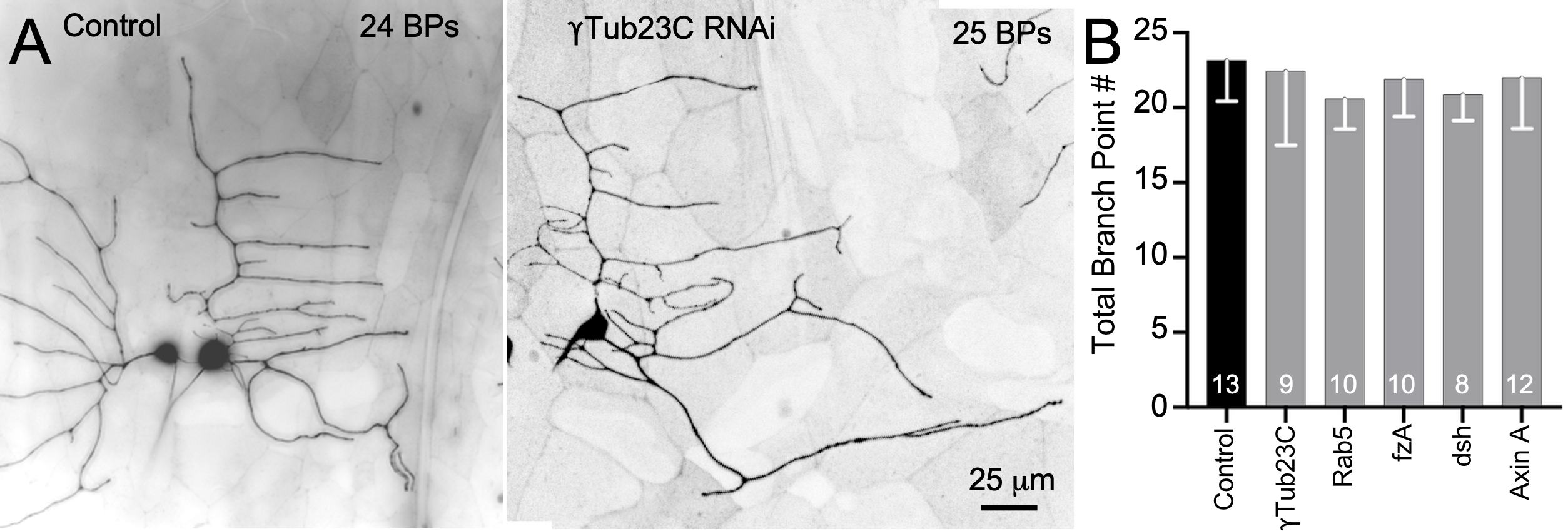

Supplement: S3 Fig — (A) Example images of class I da neurons expressing either control or gTub23C RNAi hairpins. The number of total dendrite branch points for each example is provided. (B) Quantification of total branch point number for RNAi conditions. Sample size is shown in the bars and represents number of cells quantified for each condition. Branch point number was summed between all dendritic processes, and a linear regression was used to determine statistical significance. *p < 0.05, **p < 0.01, ***p < 0.001. Refer to S1 Table for all genotypes and S1 Data for data used to generate the graph in (B). da neuron, dendritic arborization neuron; RNAi, RNA interference. (TIF) [file pbio.3000647.s003.tif]

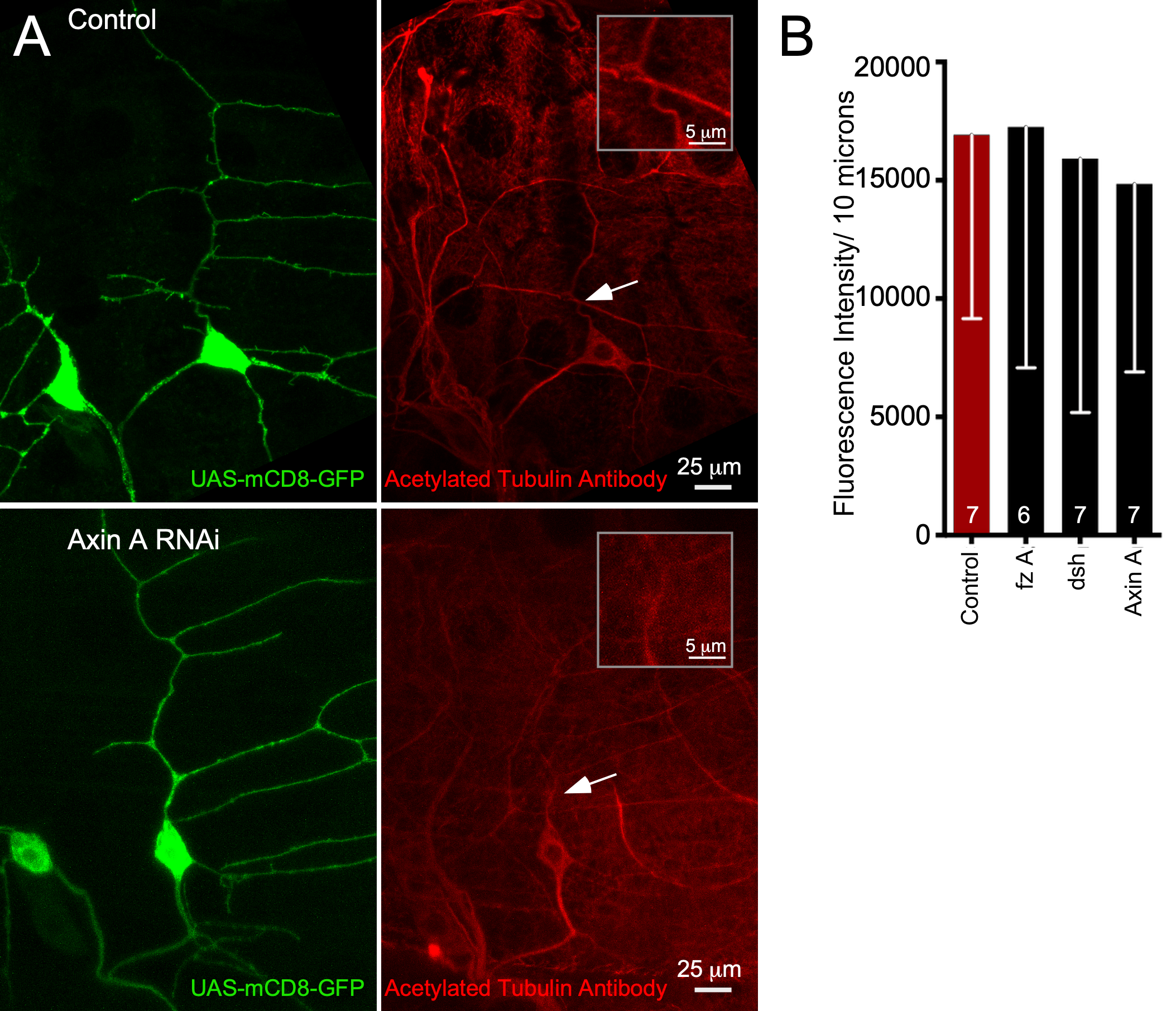

Supplement: S4 Fig — (A) Example images from filleted larva, immunostained with an antibody against acetylated tubulin (right panels). Cell shape marker is the membrane marker UAS-mCD8-GFP (left panels). The top example is control, and the bottom is Axin A RNAi. A white arrow in the red panel indicates a 10-μm region that was used for quantification of acetylated tubulin fluorescence. The inset in each panel is an enlarged view of this region. (B) Quantification of fluorescence intensity of acetylated tubulin in the first proximal 10 μm of the comb dendrite. Sample size is shown in the bars and represents the number of cells quantified. A linear regression was used to determine statistical significance. *p < 0.05, **p < 0.01, ***p < 0.001. Refer to S1 Table for all genotypes and S1 Data for data used to generate the graph in (B). GFP, green fluorescent protein; RNAi, RNA interference; UAS, upstream activating sequence. (TIF) [file pbio.3000647.s004.tif]

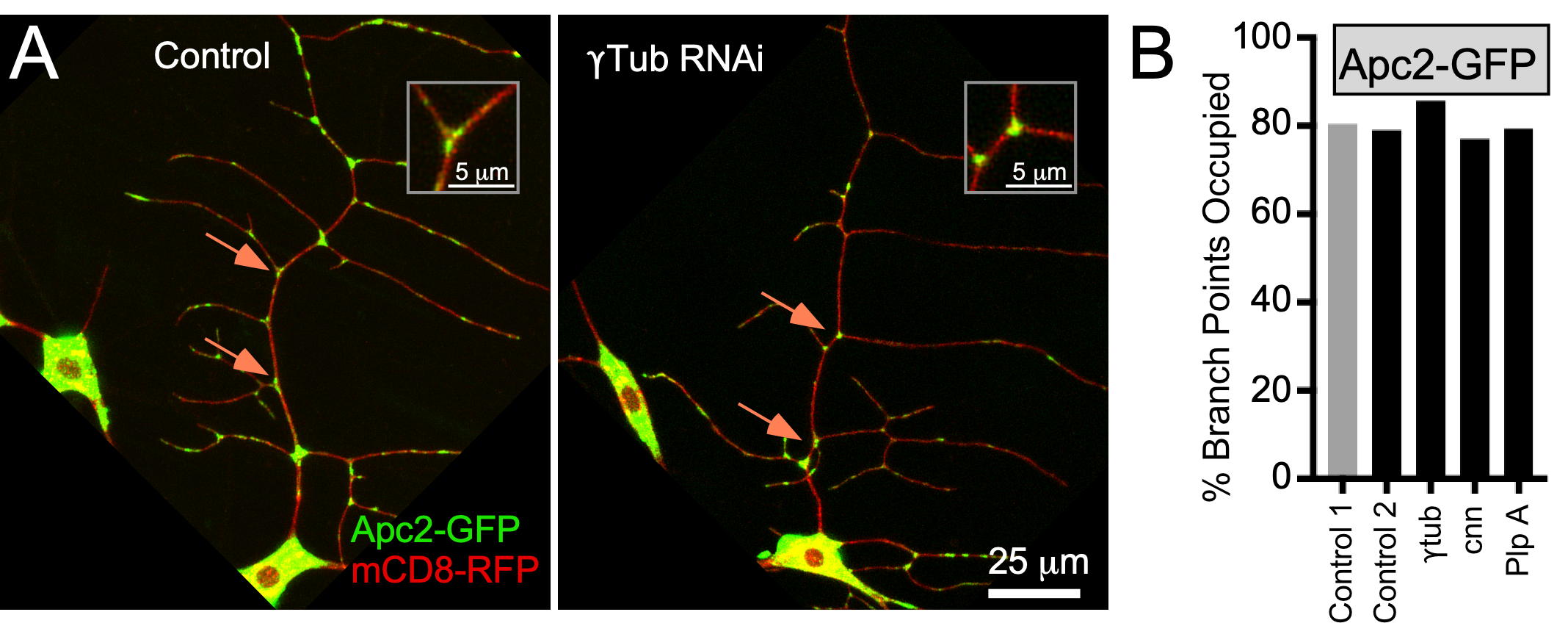

Supplement: S5 Fig — (A) Example image of Apc2-GFP and mCD8-RFP in ddaE neurons expressing UAS-Rtnl2 RNAi (control 1) (VDRC 33320) and UAS-γTub23C RNAi (VDRC 19130) hairpins are shown. Orange arrows indicate branch points with high Apc2-GFP signal, scored as occupied. Insets in the top corner of each image indicate the top branch point, indicated by an arrow. (B) Quantification of Apc2-GFP branch point occupancy is shown in neurons expressing different RNAi hairpins. Refer to S1 Table for all genotypes and S1 Data for data used to generate the graph in (B). γTub, γTubulin; Apc, adenomatous polyposis coli; cnn, centrosomin; dda, dorsal dendritic arborization; GFP, green fluorescent protein; Plp, Pericentrin-like protein; RFP, red fluorescent protein; RNAi, RNA interference; Rtnl2, reticulon 2; UAS, upstream activating sequence; VDRC, Vienna Drosophila Resource Center. (TIF) [file pbio.3000647.s005.tif]

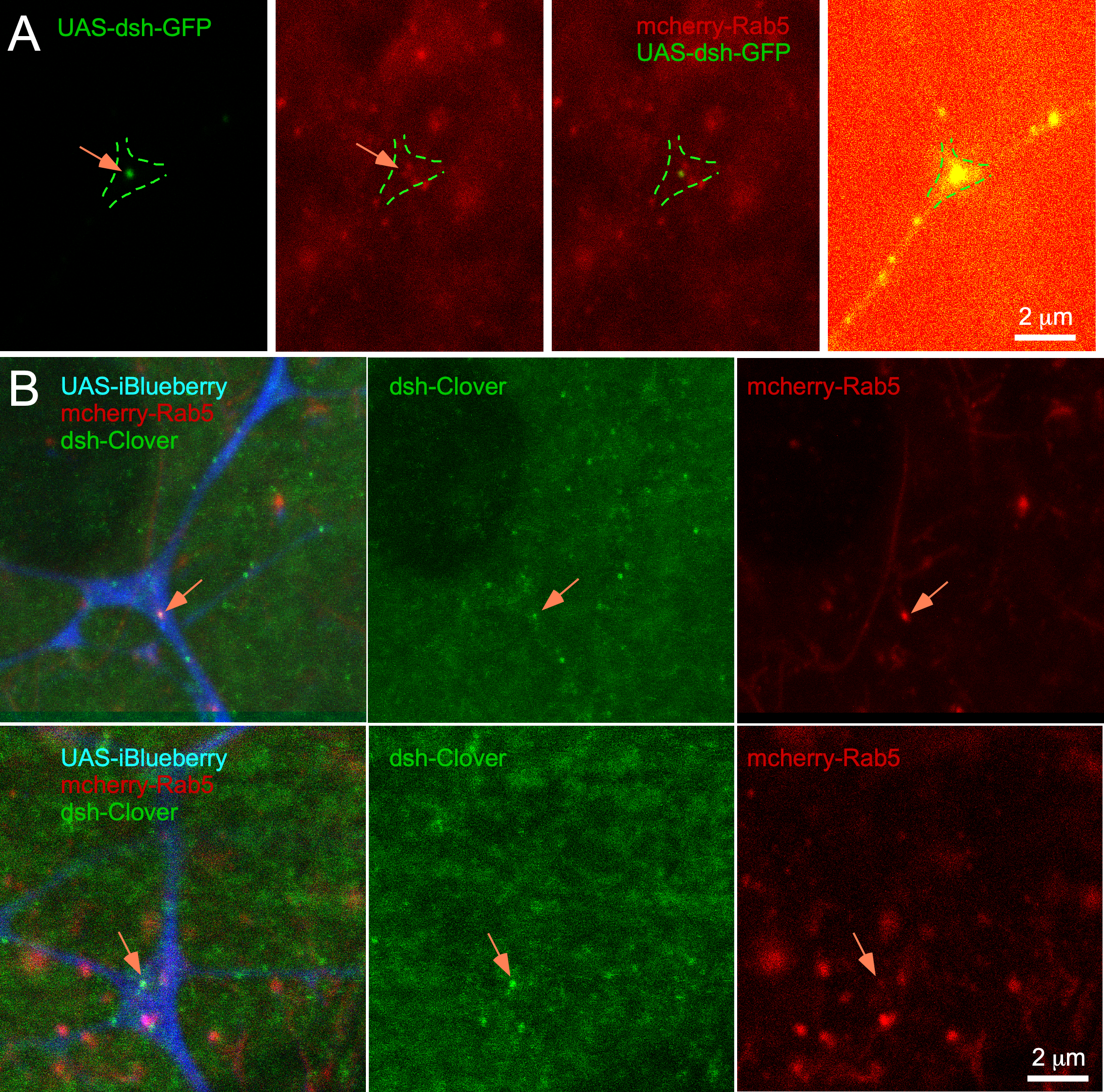

Supplement: S6 Fig — (A) Example image of endogenous mcherry-Rab5 and UAS-dsh-GFP. Because there is no membrane marker, the panel on the right was enhanced and used as a template to draw the outline of the dendrite branch point. (B) Two example images of a section of dendrite where the colocalization between mcherry-Rab5 and dsh-Clover can be seen in the branch point of the neuron. The orange arrow indicates the colocalization. The middle and right panels show the green and red channels, with orange arrows indicating colocalization. dsh, dishevelled; GFP, green fluorescent protein; UAS, upstream activating sequence. (TIF) [file pbio.3000647.s006.tif]

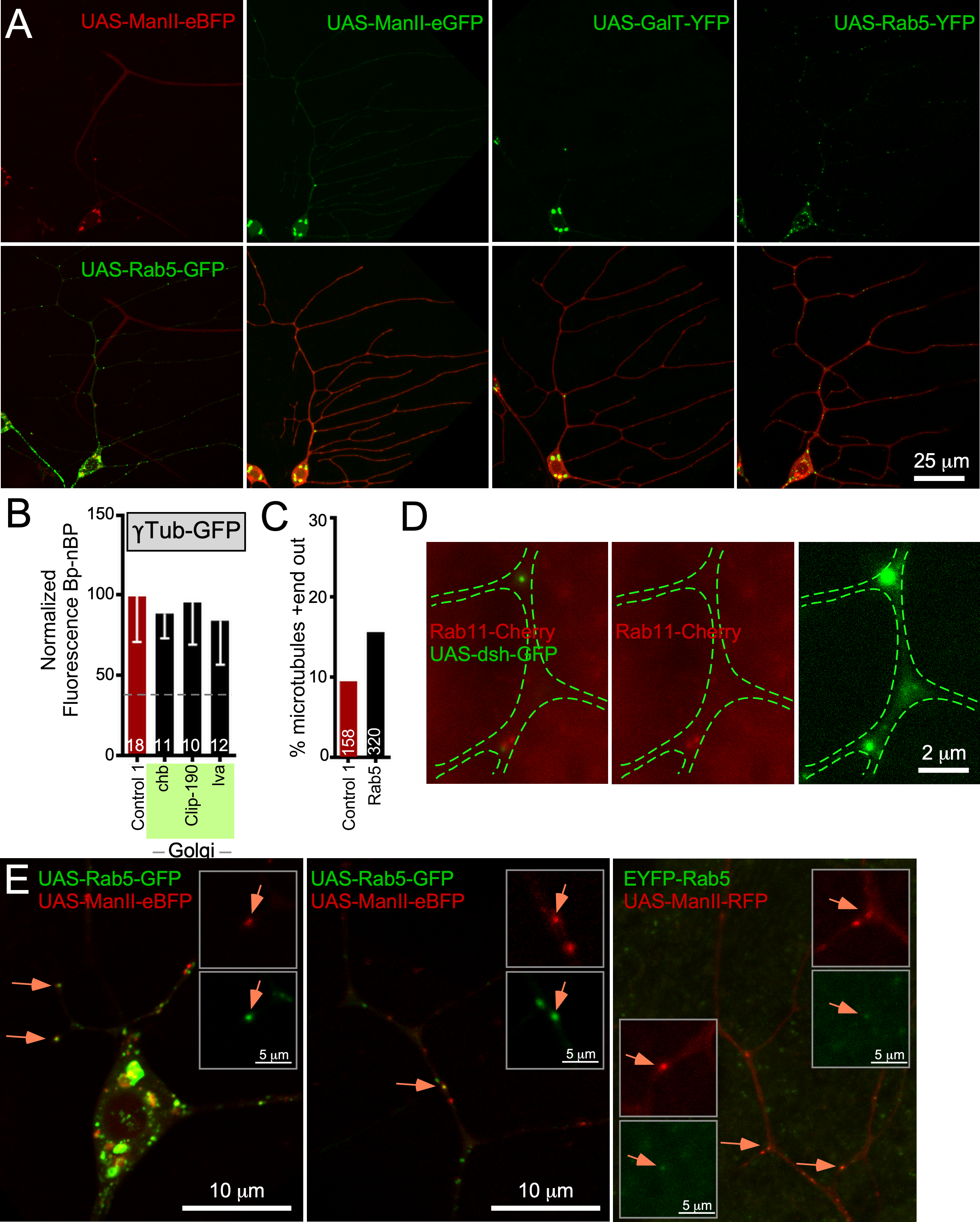

Supplement: S7 Fig — (A) Three examples of dendritic localization of Golgi markers, including UAS-ManII-eBFP, UAS-ManII-eGFP, and UAS-GalT-YFP, and one example of the early endosomal marker UAS-Rab5-YFP. The UAS-ManII-eBFP (pseudocolored) is coexpressed with UAS-Rab5-GFP. All other markers have the cell shape marker UAS-mCD8-RFP coexpressed for localization reference. (B) Quantification of γTub-GFP at BPs is shown in larvae expressing different RNAi hairpins targeting Golgi-associated proteins. Values were generated by subtracting mean nBP fluorescence from BP fluorescence for each cell; normalized fluorescence values are shown. The number of cells (one per animal) is shown on the bars. Error bars indicate standard deviation. (C) Quantification of EB1-GFP comet direction in the main trunk of the ddaE dendrite in animals expressing hairpin RNAi’s. The percentage of microtubules oriented plus-end-out is plotted as a summed value across all cells for each genotype. The numbers on each bar are total EB1-GFP comets counted, and at least 15 cells were analyzed for each genotype, with one cell per animal. A logistic regression was used to determine significance. *p < 0.05, **p < 0.01, ***p < 0.001. (D) Example image of endogenous Rab11-cherry and UAS-dsh-GFP. Because there is no membrane marker, the panel on the right was enhanced and used as a template to draw the outline of the dendrite branches. (E) Examples images of either EYFP-Rab5 or UAS-Rab5-GFP coexpressed with UAS-ManII-eBFP. The orange arrows point to puncta of colocalization between the two markers in each case. Insets in the top corner of each image show the example highlighted with an arrow in each image. For EYFP-Rab5, the bottom arrow correlates with the bottom left insets. Refer to S1 Table for all genotypes and S1 Data for data used to generate graphs in (B) and (C). γTub, γTubulin; BP, branch point; dda, dorsal dendritic arborization; dsh, dishevelled; EB1, end-binding protein 1; eGFP, enhanced green fluorescent protein; EYF [file pbio.3000647.s007.tif]

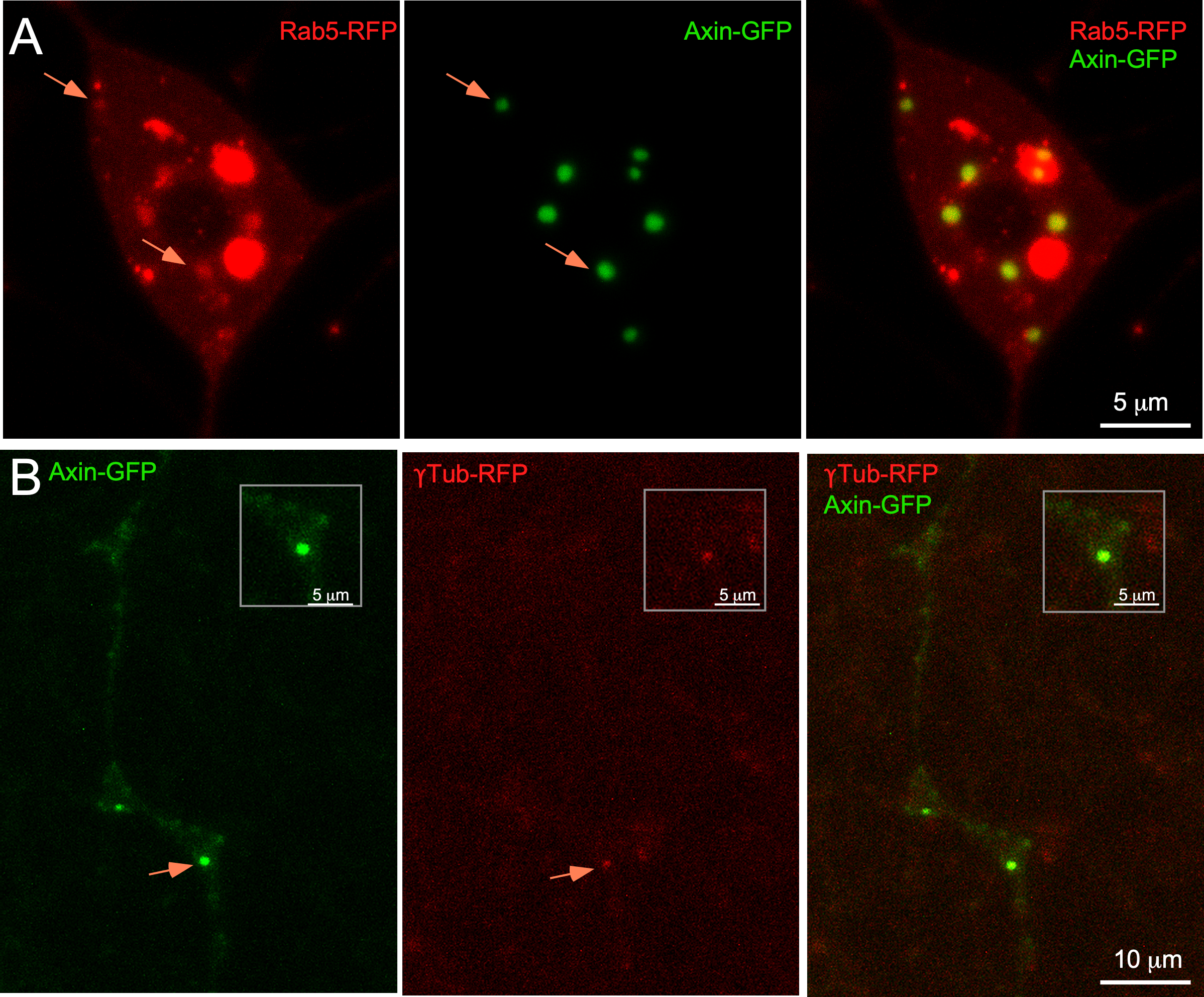

Supplement: S8 Fig — (A) Images showing the localization of UAS-Rab5-RFP in the cell body of a ddaE neuron when coexpressed with UAS-Axin-GFP. Orange arrows indicate the subset of Rab5 endosomes that colocalize with Axin-GFP. (B) Images show the localization of UAS-γTub-RFP (middle) in the branch points of a ddaE neuron when coexpressed with UAS-Axin-GFP (left) using the 221-Gal4 driver. Merged channel is provided on the right. Orange arrow points to a colocalized puncta at the branch point and is enlarged in the inset. γTub, γTubulin; dda, dorsal dendritic arborization; GFP, green fluorescent protein; RFP, red fluorescent protein; UAS, upstream activating sequence. (TIF) [file pbio.3000647.s008.tif]

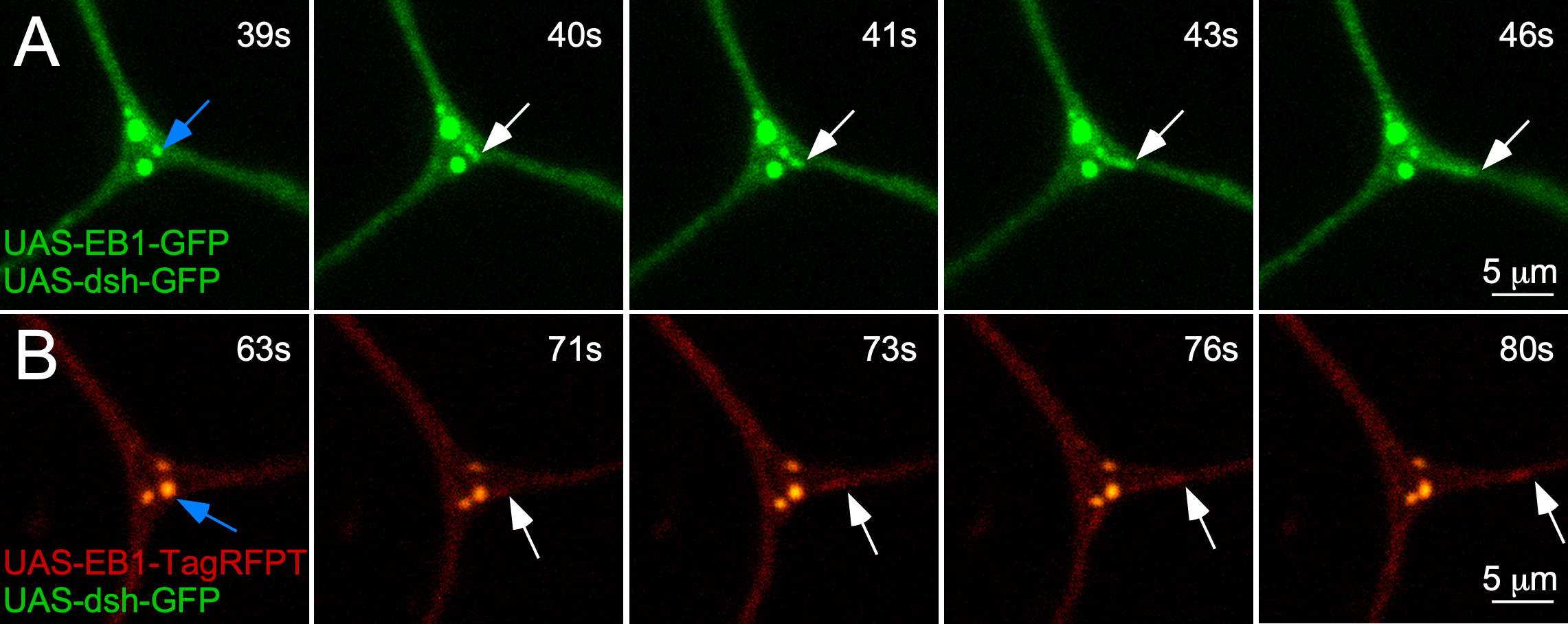

Supplement: S9 Fig — (A) Example five-frame stills of microtubule comet formation off UAS-dsh-GFP puncta. The top example is shown coexpressed with UAS-EB1-GFP, and the bottom is with UAS-EB1-TagRFPT. In both examples, the first frame includes a blue arrow to show the dsh-GFP puncta that the microtubule comet will initiate off. Subsequent frames track movement of the microtubule with a white arrow. Time stamp at the top-right corner correlates to the time point in the corresponding S8 and S12 Movies. dsh, dishevelled; EB1, end-binding protein 1; GFP, green fluorescent protein; UAS, upstream activating sequence. (TIF) [file pbio.3000647.s009.tif]

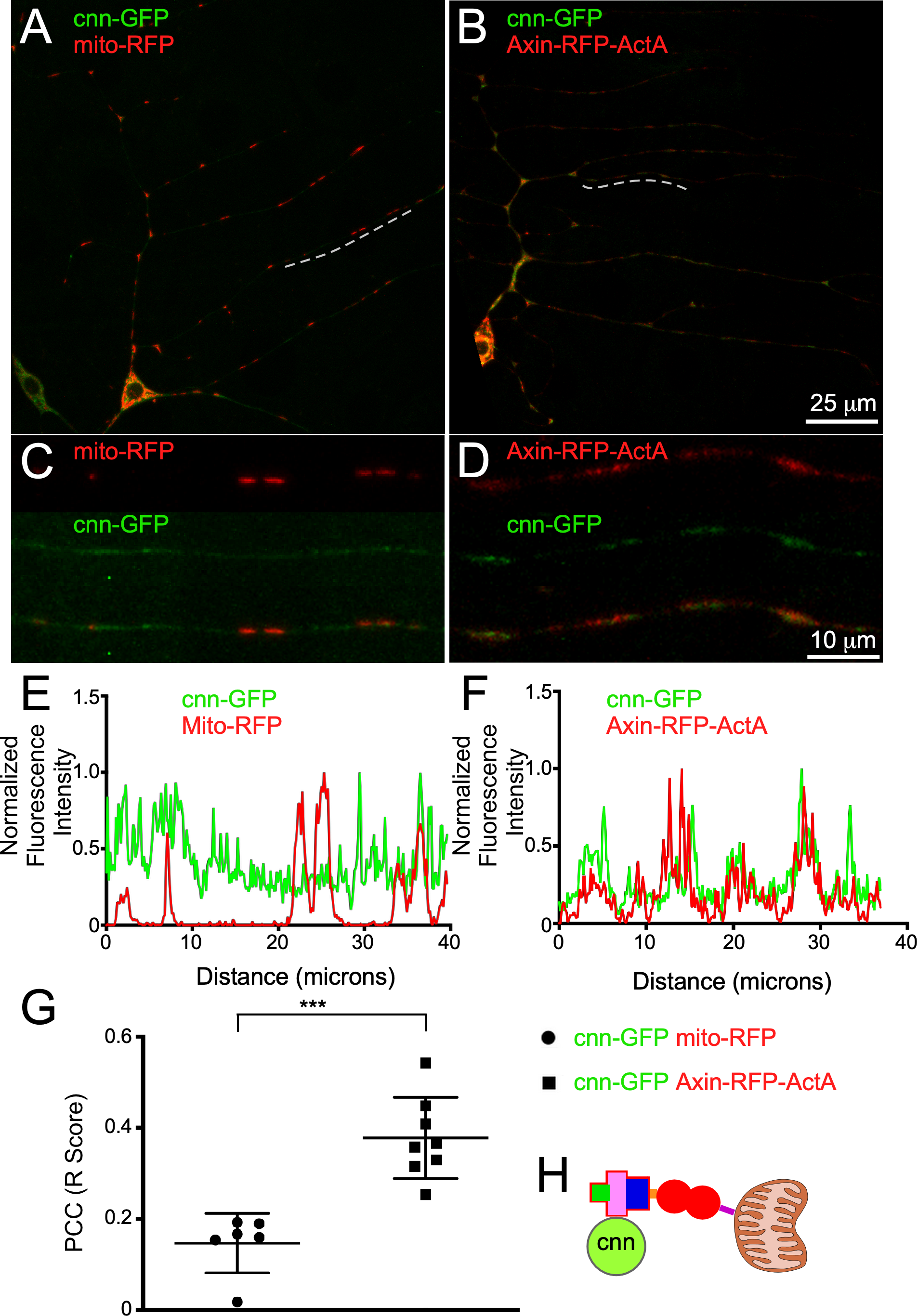

Supplement: S10 Fig — (A and B) UAS-cnn-GFP was coexpressed with either UAS-mito-RFP or UAS-Axin-RFP-ActA using 221-Gal4. Overview images of the entire dendrite arbor are shown. (C and D) Regions within the comb dendrite indicated by the dashed lines in (A) and (B). (E and F) Fluorescence intensity measurements from the regions shown in (C) and (D). (G) A plot of Pearson’s correlation coefficient between the two conditions. The y-axis indicates the R score, with 1 being positive correlation, 0 meaning no correlation, and −1 meaning negative correlation. The key to the right of the graph indicates which conditions match the symbols. (H) A diagram of the chimeric protein used to tag Axin with RFP (tdimer2[12]) and target it to mitochondria is shown. Refer to S1 Table for all genotypes and S1 Data for data used to generate graphs in (E), (F), and (G). ActA, actin assembly promoting protein A; cnn, centrosomin; GFP, green fluorescent protein; Mito, mitochondrial; RFP, red fluorescent protein; UAS, upstream activating sequence. (TIF) [file pbio.3000647.s010.tif]

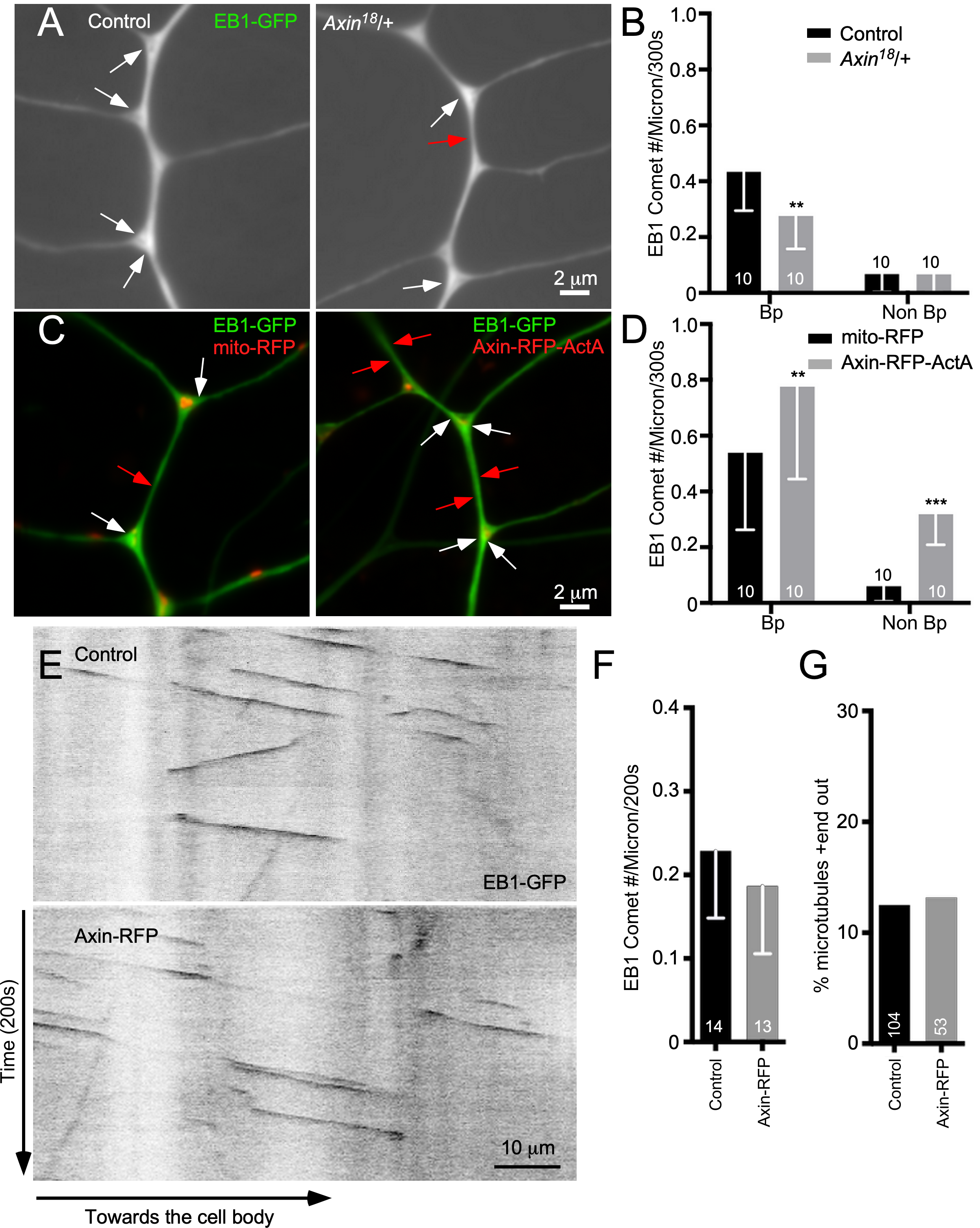

Supplement: S11 Fig — (A) Example images compiled from EB1-GFP movies of the main comb dendrite trunk were generated using a summed projection of all 300 frames. Movies were acquired in control (yw) and Axin18/+ mutant backgrounds. White arrows indicate spawning events at branch points, and red arrows indicate comets that spawn from non–branch point regions during the 300-second movie. (B) Quantification of number of EB1 comets per micrometer for the entire 300-second movie is shown. (C) UAS-EB1-GFP was coexpressed with either RFP-tagged mitochondria or the chimeric UAS-Axin-RFP-ActA. Summed example images are shown. Arrows follow the same scheme as the top two panels. (D) Quantification of number of EB1 comets per micrometer for the entire 300-second movie is shown. (E) Kymographs generated from neurons expressing either dsh-clover and UAS-EB1-GFP or UAS-Axn-RFP and UAS-EB1-GFP. dsh-clover is used as the control because it is endogenously tagged and thus is not overexpressed like the Axin-RFP. (F and G) Microtubule polarity and dynamics of control versus UAS-Axin-RFP. Sample size shown in or above the bars represents the number of cells imaged; except in the case of polarity, it is the number comets. Error bars indicate standard deviation. A linear regression was used for dynamics, and a logistic regression was used for polarity to determine statistical significance. *p < 0.05, **p < 0.01, ***p < 0.001. Refer to S1 Table for all genotypes and S1 Data for data used to generate graphs in (B), (D), (F), and (G). ActA, actin assembly promoting protein A; Axn, Axin; dsh, dishevelled; EB1, end-binding protein 1; GFP, green fluorescent protein; RFP, red fluorescent protein; UAS, upstream activating sequence; yw, yellow, white. (TIF) [file pbio.3000647.s011.tif]
